# Cell-type-resolved quantitative proteomics map of interferon response against SARS-CoV-2

**DOI:** 10.1016/j.isci.2021.102420

**Published:** 2021-04-20

**Authors:** Elisa Saccon, Xi Chen, Flora Mikaeloff, Jimmy Esneider Rodriguez, Laszlo Szekely, Beatriz Sá Vinhas, Shuba Krishnan, Siddappa N. Byrareddy, Teresa Frisan, Ákos Végvári, Ali Mirazimi, Ujjwal Neogi, Soham Gupta

**Affiliations:** 1 Division of Clinical Microbiology, Department of Laboratory Medicine, Karolinska Institutet, ANA Futura, Campus Flemingsberg, 14152 Stockholm, Sweden; 2 Division of Chemistry I, Department of Medical Biochemistry and Biophysics, Karolinska Institutet, Stockholm, Sweden; 3 Division of Pathology, Department of Laboratory Medicine, Karolinska Institutet, Stockholm, Sweden; 4 Department of Pharmacology and Experimental Neuroscience, University of Nebraska Medical Center, Omaha, NE, USA; 5 Public Health Agency of Sweden, Solna, Sweden; 6 Department of Molecular Biology and Umeå Centre for Microbial Research (UCMR), Umeå University, Umeå, Sweden

**Keywords:** Virology, Cell Biology, Proteomics

## Abstract

The commonly used laboratory cell lines are the first line of experimental models to study the pathogenicity and performing antiviral assays for emerging viruses. Here, we assessed the tropism and cytopathogenicity of the first Swedish isolate of SARS-CoV-2 in six different human cell lines, compared their growth characteristics, and performed quantitative proteomics for the susceptible cell lines. Overall, Calu-3, Caco2, Huh7, and 293FT cell lines showed a high-to-moderate level of susceptibility to SARS-CoV-2. In Caco2 cells, the virus can achieve high titers in the absence of any prominent cytopathic effect. The protein abundance profile during SARS-CoV-2 infection revealed cell-type-specific regulation of cellular pathways. Type-I interferon signaling was identified as the common dysregulated cellular response in Caco2, Calu-3, and Huh7 cells. Together, our data show cell-type specific variability for cytopathogenicity, susceptibility, and cellular response to SARS-CoV-2 and provide important clues to guide future studies.

## Highlights

• Calu-3, Caco2, Huh7, and 293FT cells are differently susceptible to SARS-CoV-2
• Caco2 shows high virus production without any prominent cytopathic effect
• Quantitative proteomics shows cell-type specific variations in cellular response
• Interferon response is commonly dysregulated in Calu-3, Caco-2, and Huh7

## Graphical abstract

**Figure:**
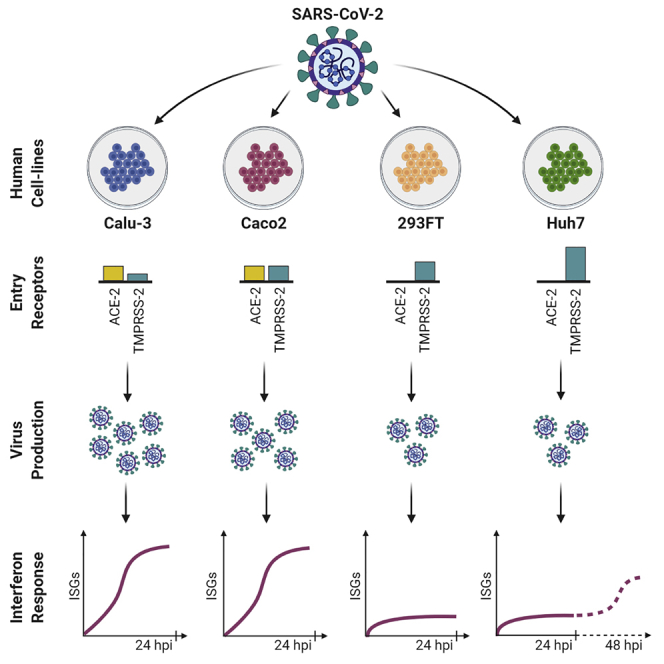

## Introduction

Severe acute respiratory syndrome coronavirus 2 (SARS-CoV-2), the causative agent of the coronavirus disease 2019 (COVID-19) pandemic, is a highly pathogenic coronavirus that has created a global public health challenge (Hu et al., 2020). The virus primarily attacks the lung, and the patients often present with severe respiratory distress. However, apart from respiratory symptoms, involvement of other organs with cardiovascular, gastrointestinal, liver, neurological, hematological, and skin manifestations in the disease pathology has been documented, suggesting the vulnerability of these anatomical sites to this virus (Gavriatopoulou et al., 2020; Trypsteen et al., 2020). This has been attributed to the presence of the primary receptor of the virus, angiotensin-converting enzyme 2 (ACE2), throughout the body (Hamming et al., 2004). However, we still lack information on the basic virology and the pathogenesis of the virus in different organs.

Three-dimensional (3D) organotypic cultures can mimic different organs of interest and allows to study SARS-CoV-2 infection in a more physiological context (Clevers, 2020; Sridhar et al., 2020). However, generating 3D organoids is technically challenging and expensive, and thus cell models are commonly employed as a simple and fast solution to study viral infection. Vero-E6 cell line that originates from monkey kidney has been widely employed for SARS-CoV-2 isolation, propagation, and antiviral testing, due to its high virus production and a prominent cytopathic effect (CPE) upon infection. Several cell lines of human origin such as Caco2, Calu-3, Huh7, and 293T were also found to be susceptible to SARS-CoV-2 (Chu et al., 2020). Numerous research activities have employed these SARS-CoV-2 susceptible cell lines to understand the mechanisms of viral pathogenesis and develop effective antivirals against the infection (Appelberg et al., 2020; Bojkova et al., 2020; Chu et al., 2020; Jureka et al., 2020; Zecha et al., 2020). Many of the studies have applied proteomics strategies to investigate the changes in the host cellular environment and the mechanism of virus-mediated re-wiring of different signaling pathways (Lachén-Montes et al., 2020). *In vitro* studies using cell models such as Caco2, Huh7, Hek293, A549, and Vero-E6 cell lines have identified interactome of different SARS-CoV-2 proteins (Gordon et al., 2020; Li et al., 2021; Stukalov et al., 2020) as well as measured the changes in global protein abundance over time following infection including ours (Appelberg et al., 2020; Bojkova et al., 2020; Zecha et al., 2020). Variance in infectivity of cell lines was noted between studies. One of the first quantitative proteomics study by Bojkova et al. showed high infectivity and cytopathogenicity in Caco2 infected with the Frankfurt strain (Bojkova et al., 2020), whereas in another study by Zecha et al. the Munich strain did not show infectivity in Caco2 cells (Zecha et al., 2020). Vero-E6 has also been used as a cell model in several proteomics studies (Grenga et al., 2020; Zecha et al., 2020). However, Vero-E6 may not be able to recapitulate the complexity of host cellular response to the SARS-CoV-2 in humans because it originates from monkey and lacks the gene cluster associated with type-I interferon (IFN-I) (Osada et al., 2014). Among the cell lines originating from lungs the primary site of infection, SARS-CoV-2 was shown to efficiently replicate in Calu-3 cells (Banerjee et al., 2020; Chu et al., 2020; Hsin et al., 2020). However, presently no proteomics data is available from SARS-CoV-2-infected Calu-3 cells.

In this study, we show the susceptibility of the first Swedish isolate of SARS-CoV-2 in six commonly used laboratory human cell lines. Using quantitative proteomics, we determined the changes in protein abundance caused by the virus in the susceptible cell lines, providing an overview of the signaling pathways that are altered by the SARS-CoV-2.

## Results

### Susceptibility and cytotoxicity of first Swedish SARS-CoV-2 isolate in commonly used laboratory cell lines

**Figure 1:**
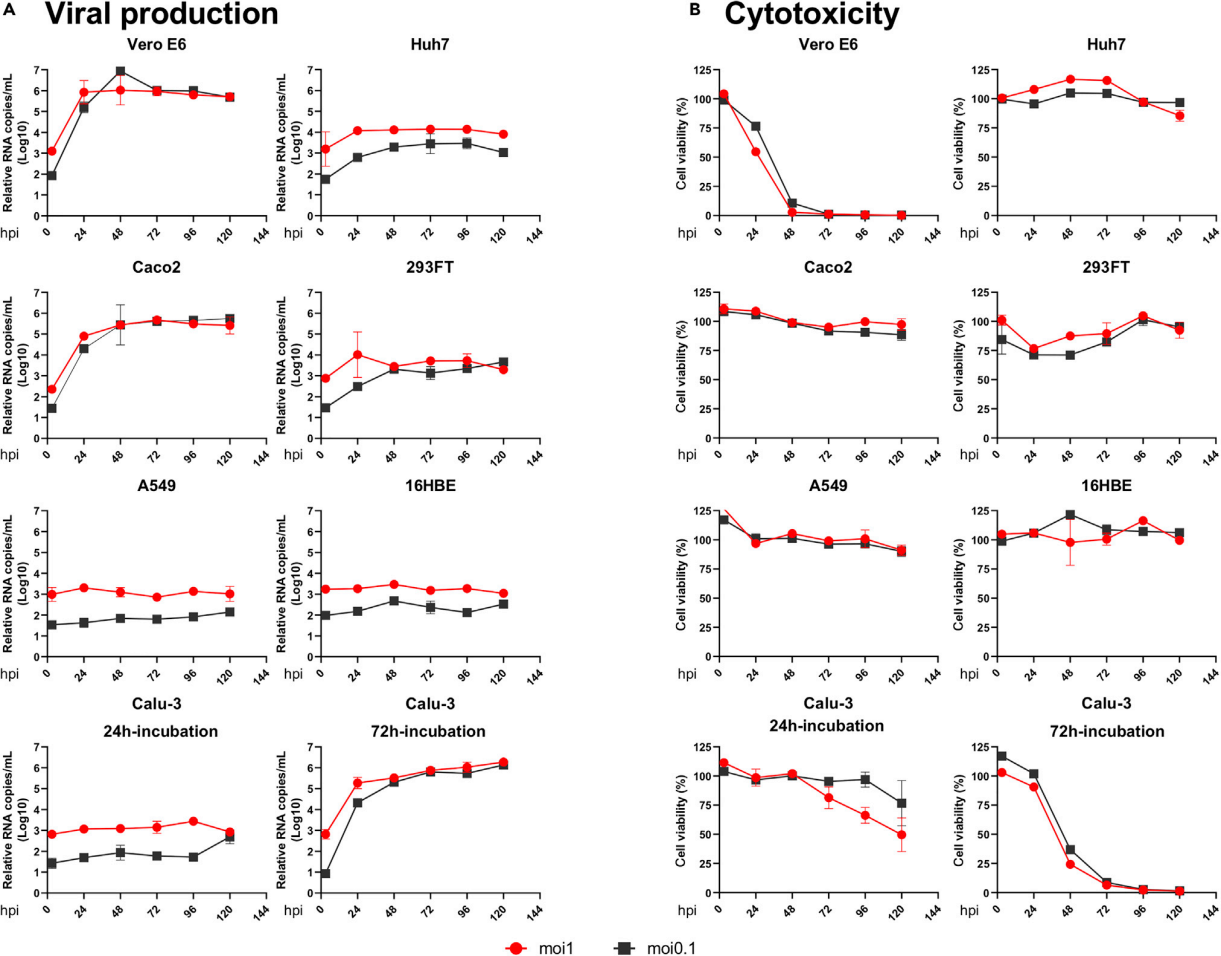
Viral production and cytopathogenicity of SARS-CoV-2 in Vero-E6 and six different cell lines of human origin Indicated cell lines were infected with SARS-CoV-2 at moi of 1 and 0.1 either in duplicate or triplicate. (A) Viral supernatant samples were harvested at 3hpi, 24hpi, 48hpi, 72hpi, 96hpi, and 120hpi. Viral production was determined by qRT-PCR targeting the N gene of SARS-CoV-2 comparing each time point with 3hpi. (B) Cell viability was measured at 3 h post infection (hpi), 24hpi, 48hpi, 72hpi, 96hpi, and 120hpi by Viral-ToxGlo assay. The viability at each time was determined in comparison to the uninfected control.

We infected Vero-E6, Calu-3, A549, Caco2, Huh7, 293FT, and 16HBE with the first Swedish isolate of SARS-CoV-2 virus (SWE/01/2020) at a multiplicity of infection (moi) of 1 and 0.1 as previously described (Appelberg et al., 2020). Virus-induced cytotoxicity was evaluated by measuring the cellular ATP using Viral-ToxGlo assay (Promega), and virus production was determined by measuring the presence of viral genome in the cell culture supernatant using qPCR to determine the N-gene RNA (Corman et al., 2020) starting at 3-h post-infection (hpi) and followed-up to 120hpi (Appelberg et al., 2020). As shown in Figure 1A, infection with moi 0.1 or 1 showed a very similar pattern of virus production over time and by the end of 120 h attained similar viral copies in the supernatant. Infection at moi 0.1 induced significant cytopathogenicity in Vero-E6 (less than 3% viability by 48hpi) and a significant increase of 3.8 log10 viral RNA copies in the supernatant at 24hpi. Of the six human cell lines that were tested, Caco2 (intestinal) and Calu-3 (lung) that were seeded for 72 h prior to infection showed the highest virus production with >4 log10 RNA copies by 48hpi (p < 0.001) and thereafter marginal increase till 120hpi. It was interesting to note that Calu-3 cells, which were infected after 72 h of seeding and showed tightly closed together cells with polygonal or cuboidal features and defined boundaries, had a higher susceptibility to SARS-CoV-2 compared with Calu-3 cells that were infected after 24 h of seeding (round and isolated) (Figure S1A). Immunofluorescent staining for β-catenin and β-actin that are essential for the organization of polarized epithelium and cell-to-cell contact showed a defined co-localization along the cell margin after 72 h of incubation, indicating polarization of the Calu-3 cells (Figure S1B). This leads us to speculate that enhanced susceptibility of Calu-3 with longer incubation of 72 h prior to infection was possibly due to polarization of the cells (Foster et al., 2000), as it was reported previously for SARS-CoV (Tseng et al., 2005). 293FT (kidney; p < 0.01) and Huh7 (liver; p < 0.02) showed moderate virus production with >1 log10 viral RNA copies in the supernatant by 120hpi. 16HBE (lung) and A549 (lung) cells showed very poor virus production with <0.6 log10 RNA copies. Interestingly, other than Vero-E6, viral-induced cytotoxicity was only observed in Calu-3 cells with a viability of ≤50% by 48hpi and ≤80% by 72hpi. None of the other cell lines showed any apparent cytotoxicity (viability>85%) (Figure 1B).

**Figure 2:**
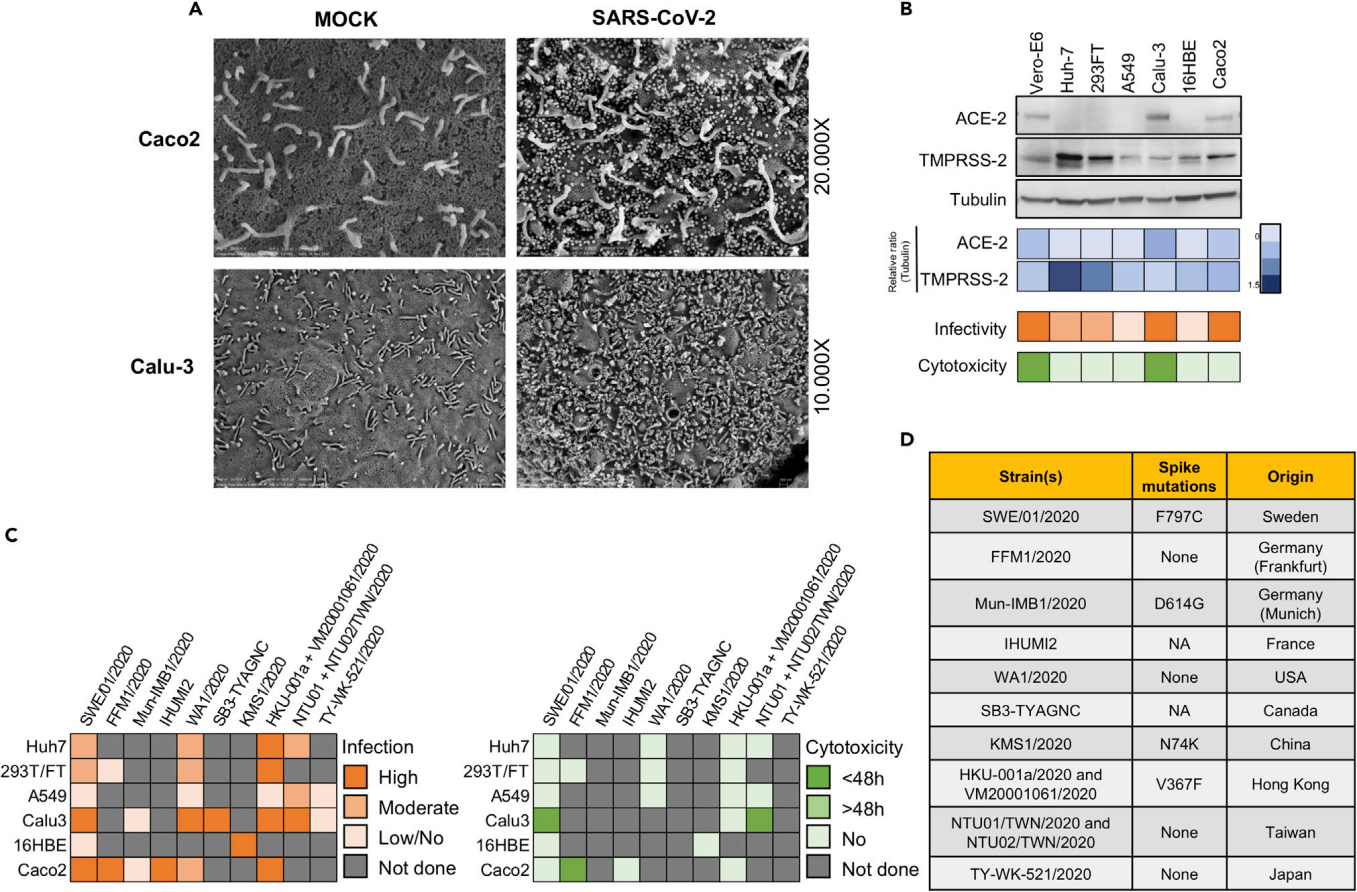
Cell-specific and strain-specific differences in SARS-CoV-2 tropism (A) Scanning electron microscopy images of the cell surface of mock-infected and SARS-CoV-2-infected (moi 0.1) Caco2 and Calu-3 cells. Indicated cell lines were infected with SARS-CoV-2 at moi of 0.1 and fixed at 48hpi for microscopy analysis. (B) ACE2 and TMPRSS2 receptor expression in six human cell lines. ACE2 and TMPRSS2 expression in the indicated cell lines was determined by western blot in the cell lysates. Heatmaps with relative protein quantification (blue), susceptibility (orange), and cytotoxicity (green) are shown. (C) Susceptibility of globally isolated SARS-CoV-2 strains. Based on previously published or preprint articles, a chart was created showing the susceptibility of the commonly used laboratory cell lines and cytopathogenicity of SARS-CoV-2 strains isolated from different geographical locations. Boxes are color coded based on intensity as indicated in the figure. The moi used wherever available is mentioned in the Table S2. (D) Table showing the presence of any amino acid substitution in the spike protein of the indicated strains. NA = not available.

We observed that the virus production in susceptible cell lines reached saturation by 48hpi. Therefore, we investigated the changes caused in the cell surface of Calu-3, Caco2, Huh7, and 293FT cells during virus production at 48hpi (moi 0.1) using scanning electron microscopy (SEM). We did not observe any significant changes in the morphology of the cell surface in the mock-infected cells. In SARS-CoV-2-infected Calu-3 and Caco2 cells, numerous virus-like particles corresponding to the size of SARS-CoV-2 (approx. 70nM) were observed to be attached to the cell surface or cellular projections (Figure 2A). Interestingly, even though there was moderate virus production in Huh7 and 293FT cells we did not observe any attached virus-like particles on the cell surface after scanning several fields (data not shown). The possible reasons for this could be either low-level virus production in Huh7 cells and 293FT cells compared with Calu-3 and Caco2 cells that were missed visually or the virus release mechanism that is different than the budding out of the virus (Ghosh et al., 2020).

### Expression of ACE2 and TMPRSS2 in different cell lines

Angiotensin-converting enzyme 2 (ACE2) receptor and transmembrane serine protease 2 (TMPRSS2) activity have been shown to be critical for SARS-CoV-2 entry into the cell (Jureka et al., 2020; Matsuyama et al., 2020). To correlate the ACE2 and TMPRSS2 expression with the tropism, we determined the protein expression in the cell lysates by Western blot (Figure 2B). Among the infected cell lines, ACE2 expression was only observed in Vero-E6, Calu-3, and Caco2 that correlated with high virus production. When we looked into co-expression of ACE2 and TMPRSS2, it was most prominent in Caco2, followed by Vero-E6 and Calu-3, although Calu-3 showed low TMPRSS2 expression. Contrarily, Huh7 and 293FT strongly expressed TMPRSS2 but lacked ACE2 expression, indicating that each receptor has an individual role in aiding the infection.

### Susceptibility and cytopathogenicity of globally isolated SARS-CoV-2 strains

To compare the tropism of the Swedish SARS-CoV-2 virus with the other globally isolated strains, we performed a literature survey to determine the susceptibility and cytopathogenicity in all the six cell lines as noted above. We have included virus isolates from Germany (FFM1/2020) (Bojkova et al., 2020) and (Mun_IMB1/2020) (Zecha et al., 2020), France (IHUMI2) (Wurtz et al., 2021), USA (WA1/2020) (Jureka et al., 2020), Canada (SB3-TYAGNC) (Banerjee et al., 2020), China (KMS1/2020) (Liao et al., 2020), Hong Kong (HKU-001a/2020 (Chu et al., 2020) and VM20001061/2020 (Hui et al., 2020), Taiwan (NTU01/2020 and NTU02/2020) (Hsin et al., 2020), and Japan (TY-WK-521/2020) (Matsuyama et al., 2020) and found major differences in cellular tropism and cytopathogenicity (Figure 2C). Specifically, we observed that the majority of the strains were able to infect both Caco2 and Calu-3, except for the Muc_IMB1/2020 (both the cell lines not susceptible) (Zecha et al., 2020) and the Japanese strain (Calu-3 cells not susceptible) (Matsuyama et al., 2020). The Frankfurt_FFM1/2020 strain showed a rapid CPE in Caco2 by 24hpi at moi 0.1 (Bojkova et al., 2020), whereas other strains, including the Swedish isolate, did not show any prominent CPE either at higher infective dose or with prolonged time of incubation (Chu et al., 2020). In addition, using the Wuhan/Hu-1/2019 strain as a reference, we compared the amino acid changes in the spike protein of these strains. The Frankfurt and Taiwan strains showed no dissimilarities, whereas the Swedish (F797C), Munich (D614G), China (N74K), and Hong Kong (V367F) strains each showed a single amino acid substitution (Figure 2D).

### Proteomic analysis of the cell lines

**Figure 3:**
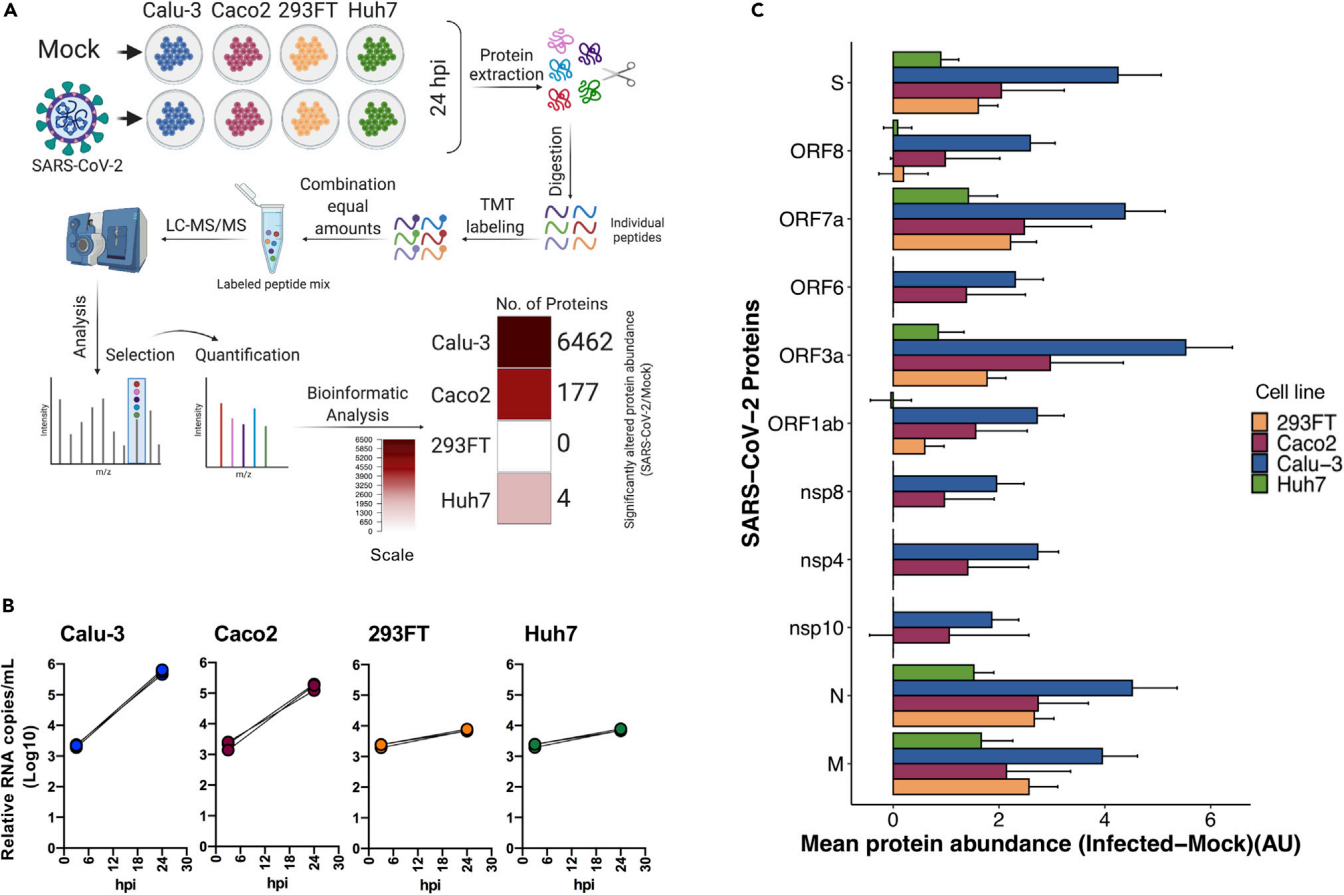
The proteomic landscape of different commonly used laboratory cell lines upon SARS-CoV-2 infection (A) Brief methodology of the proteomics experiment in the indicated cell lines that were either mock-infected or infected with SARS-CoV-2 moi 1 for 24 h. The graphical representation was created using BioRender. Significantly altered host proteins in the indicated cell lines are represented in the heatmap. (B) Relative viral production in the cell supernatant, measured at 3hpi and 24hpi. (C) Detected viral proteins in the indicated cells by tandem mass tag-labeling mass spectrometry (TMT-MS). The bars represent the difference in viral proteins between infected and uninfected cells (mean abundance of three replicates ± pooled SD) in arbitrary unit (AU). Data were prior quantile normalized.

Because we observed differential susceptibility to SARS-CoV-2 infection in different cell lines originating from the human lung (Calu-3), intestine (Caco2), liver (Huh7), and kidney (293FT), we investigated how the cellular proteins are regulated during infection in these cell lines. To this end, we either infected or mock-infected Calu-3 cells (polarized), Caco2 cells, Huh7 cells, and 293FT cells with SARS-CoV-2 (moi 1) in triplicates. For proteomics, we used 24 h of infection because at this time point despite maintaining high viability all the cell lines showed exponential virus production, that plateaued after 24hpi. The cells were harvested at 24hpi, lysed, and equal amounts of the proteins were used to perform quantitative proteomics using a TMT-labeling strategy as previously described (Appelberg et al., 2020). The unprocessed and processed raw data are presented in supplementary tables (Tables S4, S5, S6, S7, S8, S9, S10, and S11). Among the four cell lines, Calu-3 showed major changes in protein abundance upon infection, with 6,462 proteins differentially expressed in infected cells compared to the mock, followed by Caco2 with a significant difference in 177 proteins. No change in the global protein abundance was observed in Huh7 (only four proteins differentially expressed) and 293FT (no proteins differentially expressed) at 24hpi (Figure 3A, Table S12). The PCA plot showing the sample-to-sample relationship and the volcano plot showing the differentially altered protein abundance in SARS-CoV-2-infected cells as compared with the mock-infected cells are shown in Figure S2. The virus secretion in the cell culture supernatant at 3hpi and 24hpi is shown in Figure 3B. The viral protein abundance in the cells is shown in Figure 3C. The proteins that were detected are ORF1ab, ORF3a, ORF6, ORF7a, ORF8, M, N, S, nsp4, nsp8, and nsp10. The higher abundance of viral proteins detected in Calu-3 correlated with a higher level of virus production and the change in host protein abundance.

### Type-I IFN signaling is commonly dysregulated in Calu-3, Caco2, and Huh7 cell lines by SARS-CoV-2

**Figure 4:**
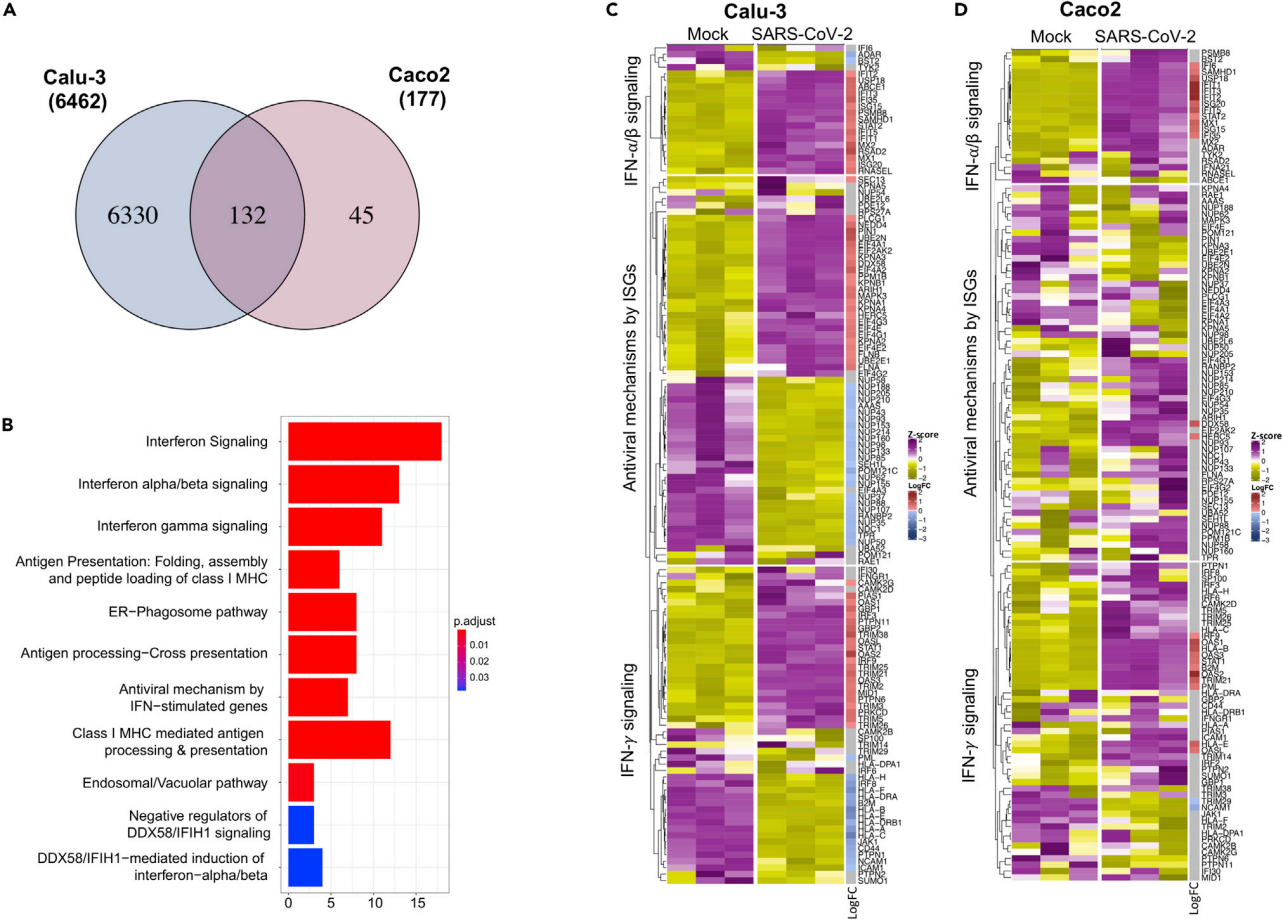
Interferon-signaling pathways are commonly dysregulated by SARS-CoV-2 infection in Caco2 and Calu-3 cell lines (A) Venn diagram and overlap of proteins with significant change in abundance between mock-infected and SARS-CoV-2-infected Caco2 and Calu-3 cell lines at 24hpi. (B) Barplot enrichment map of significant overlapping proteins in Caco2 and Calu-3 cell lines from ReactomePA. Number of proteins are indicated on the x axis. p values are indicated from highly significant in red to significant in blue. (C) Heatmap representing the number of significant proteins (LIMMA, FDR <0.05) between mock-infected and SARS-CoV-2-infected Calu-3 cell line at 24hpi. (D) Heatmap representing the number of significant proteins (LIMMA, FDR <0.05) between mock-infected and SARS-CoV-2-infected Caco2 cell line at 24hpi.

Because Calu-3 and Caco2 were the only cell lines that showed substantial protein regulation upon infection at 24 h, we compared the significantly regulated proteins in those two cell lines. As shown in the Venn diagram in Figure 4A, there were 132 proteins that were commonly dysregulated in both Calu-3 and Caco2. Among the 132 dysregulated proteins, 88 proteins were similarly upregulated (44 proteins) and downregulated (44 proteins) in both the cell lines (Figure S3). Reactome pathway analysis on the 132 significantly altered proteins common to both cell lines showed a strong enrichment of type-I and type-II interferon signaling pathways and its related RIG-I/MDA5 (DDX58/IFIH1) signaling pathway (Figure 4B). To investigate which are the proteins associated with this pathway changing upon infection, as a next step, we assessed the changes induced by the infection in the levels of each protein associated with the following pathways: interferon response, including the interferon-alpha/beta signaling (Pathway: R-HSA-909733), interferon-gamma signaling (Pathway: R-HSA-877300), and the antiviral mechanism by IFN-stimulated genes (ISGs, Pathway: R-HSA-1169410). As shown in the heatmaps, 105 (out of 129 detected) and 27 (out of 131 detected) were differentially regulated in Calu-3 (Figure 4C, Table S13) and Caco2, respectively (Figure 4D, Table S13). At 24hpi, both 293FT and Huh7 did not show any differentially regulated protein belonging to IFN-signaling pathways (109 detected proteins; Table S13) and the heat maps are shown in Figure S4. As shown in the heatmaps, among 129 detected proteins belonging to these pathways, 105 were differentially regulated in Calu-3 (63 upregulated and 42 downregulated), whereas in Caco2 among 131 detected proteins 27 were differentially regulated (25 upregulated and 2 downregulated) (Figures 4C and 4D, Table S13). The protein-protein interaction network of the significantly altered proteins showed two definite clusters in Calu-3: one including proteins associated with RIG-I (DDX58) and type-I/-II signaling complex and another majorly including components of nucleoporin complex (downregulated) and the karyopherin family (upregulated) (Figure S5A). A single cluster of proteins related to RIG-I (DDX58) and type-I signaling complex were upregulated in Caco2 (Figures S5B). In general, we observed an interferon stimulation in SARS-CoV-2-infected Calu-3 and Caco2 cells. SARS-CoV-2 receptor ACE2 has been considered to be an interferon stimulatory gene (Ziegler et al., 2020). However, we did not observe any significant differences in the protein levels of ACE2 or TMPRSS2 upon infection in our proteomics data, rather ACE2 was downregulated in SARS-CoV-2 infected Caco2 and Calu-3 cells (Figure S6).

**Figure 5:**
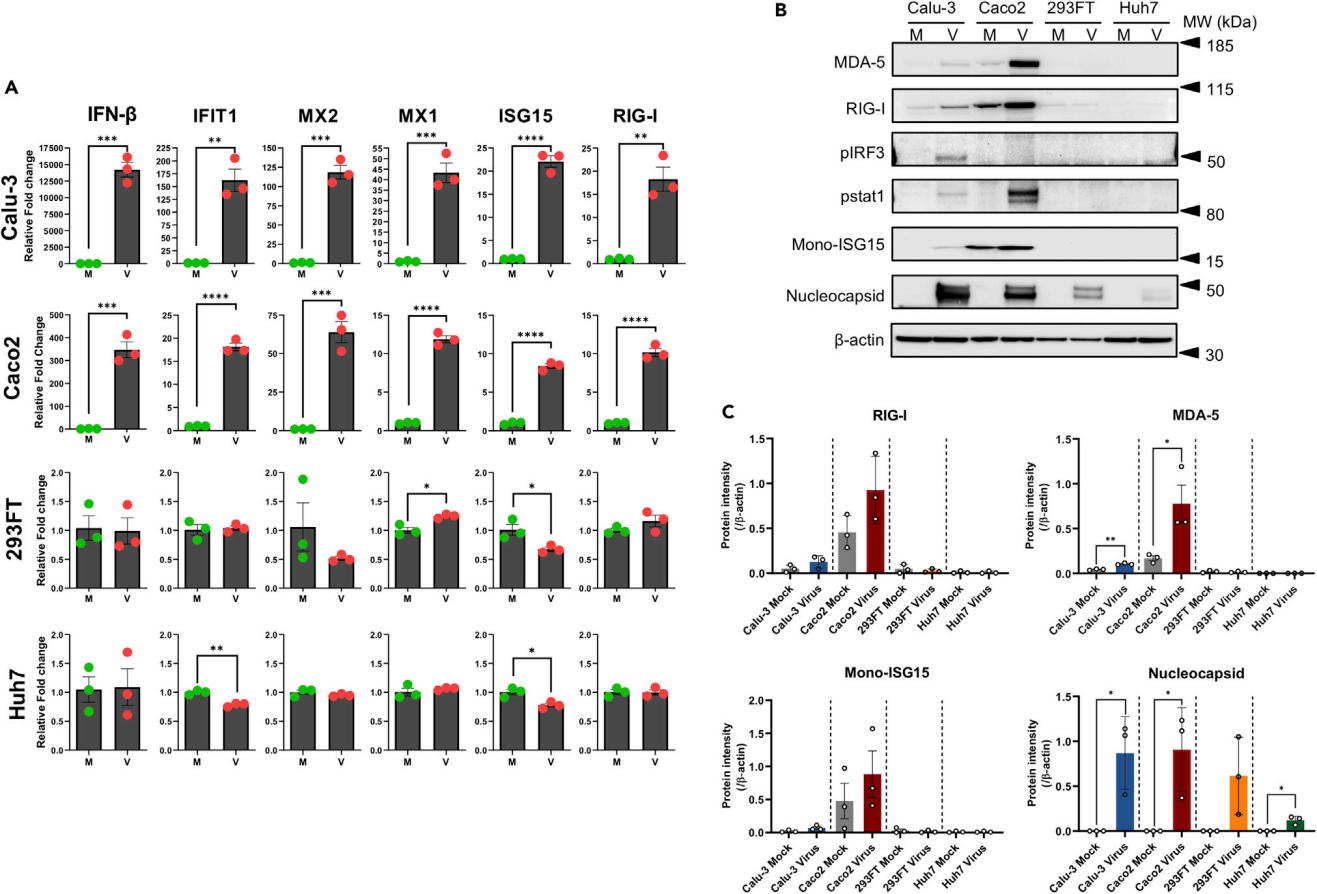
Different expression of ISGs in the infected cell lines (A) The gene expression level of IFN-β and indicated representative interferon-stimulated genes (ISGs): IFIT1, MX2, MX1, ISG15, and RIG-I in Calu-3, Caco2, 293FT, and Huh7 cell lines at 24hpi. The results are shown as fold change relative to mock-infected cells, normalized to GAPDH. The mean ± SEM of three experiments is shown. p values are determined by unpaired t test, ∗p < 0.05, ∗∗p < 0.01, ∗∗∗p < 0.001, and ∗∗∗∗p < 0.0001. M = mock; V = virus. (B) Protein expression levels of MDA-5, RIG-I, pIRF-3, p-STAT1, and viral nucleocapsid in SARS-CoV-2-infected and mock-infected Calu-3, Caco2, 293FT, and Huh7 cells at 24hpi. (C) The intensity of specific bands for MDA-5, RIG-I, mono-ISG15, and SARS-CoV-2 nucleoprotein was quantified by ImageJ and the protein intensity normalised to β-actin is represented as bars. The intensity of p-IRF3 and p-STAT1 was not quantified, as expression could be observed only in one of the experimental replicates. The mean ± SEM of three experiments is shown.

Type-I IFN, like IFN-β, plays a major role in host defenses against various viruses, and in our proteomics data we observed dysregulation of type-I-IFN-response-associated proteins including ISGs; therefore, we assessed the mRNA expression of IFN-β and the downstream ISGs (IFIT1, MX1, MX2, ISG15, and RIG-I) using qPCR. In line with our proteomics findings, the mRNA levels of IFN-β and all the tested ISGs significantly increased upon SARS-CoV-2 infection in Calu-3 and Caco2 cells. 293FT and Huh7 cells did not show any significant variations at 24hpi (Figure 5A). IFN-β is released by cells through a signaling cascade that is initiated after activation of RIG-I or RLRs upon interaction with viral products. The western blot analysis showed a major increase in activation of RIG-I, MDA-5, and its downstream effectors p-IRF3, p-STAT1, and ISG15 in infected Calu-3 and Caco2 cells (Figures 5B and 5C) corroborating the qPCR results. In general, the steady-state level of RIG-I, MDA-5, and ISG15 were higher in Caco2 and Calu-3 cells as compared with 293FT and Huh7 cells, and the latter two showed no comparable changes following 24hpi (Figure 5C). No observable change was noted in conjugated ISG15 in any of the cell lines (Figures S7A and S7B). It is conceivable that the observed changes in SARS-CoV-2-infected Calu-3 and Caco2 cells at 24hpi could be due to a high level of infectivity as observed by the expression of SARS-CoV-2 nucleoprotein (Figures 5B and 5C) and higher susceptibility (Figures 1B and 3B) as compared with 293FT and Huh7.

**Figure 6:**
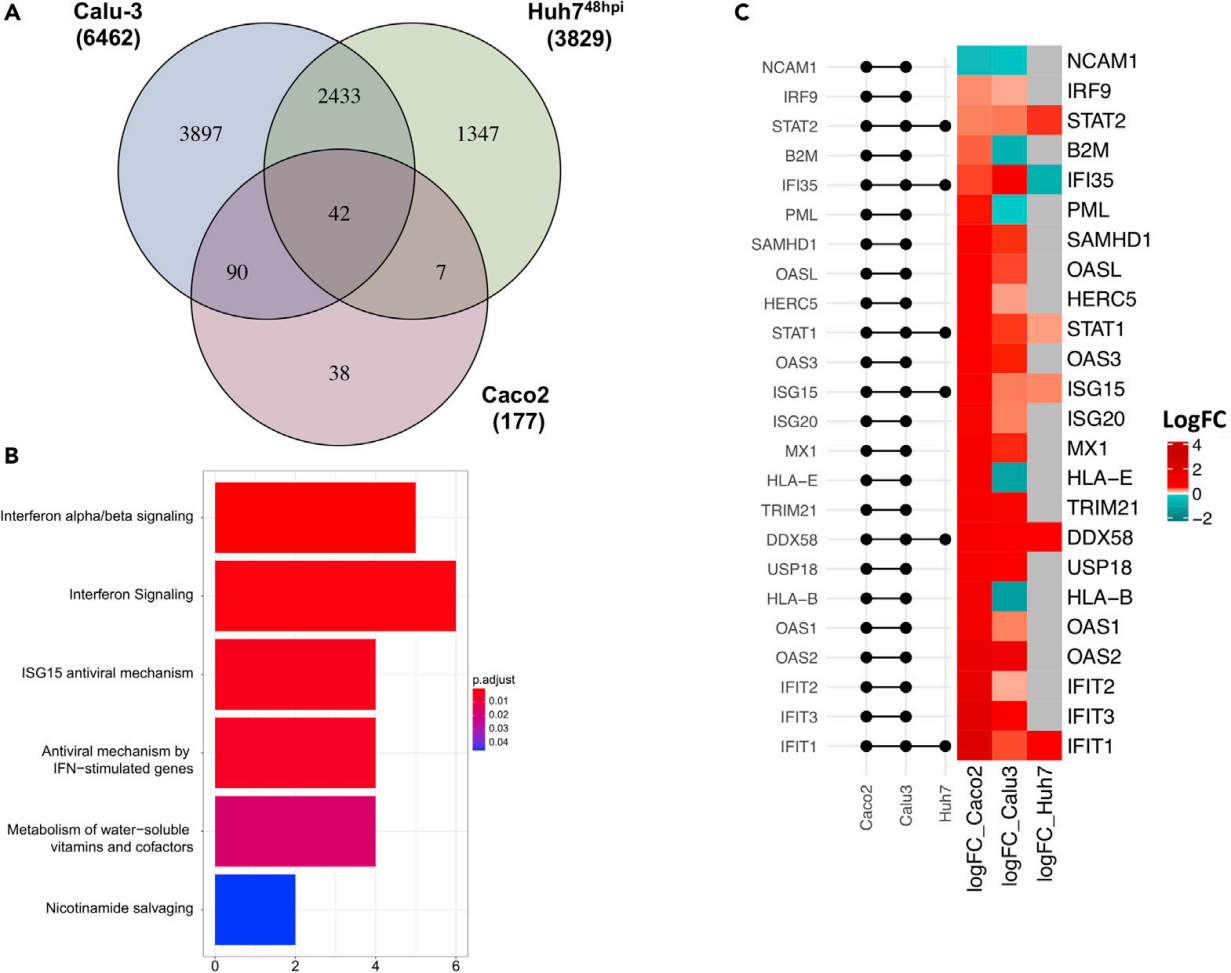
Overlap of three cell lines confirm the implication of interferon pathway in SARS-CoV-2 infection (A) Venn diagram and overlap of proteins with significant change in abundance between mock-infected and SARS-CoV-2-infected Caco2 and Calu-3 cells at 24hpi and Huh7 cells at 48hpi. (B) ReactomePA barplot enrichment map of significant overlapping proteins in Caco2, Calu-3, and Huh7 cell lines. (C) Heatmap of log fold changes of proteins associated with IFN signaling in SARS-CoV-2-infected Calu-3, Caco2, and Huh7 cells. LogFC between mock-infected and SARS-CoV-2-infected Calu-3 and Caco2 cells at 24hpi and Huh7 cells at 48hpi (right panel). Log fold changes associated with non-significant proteins are represented in gray. Log fold changes associated with significantly downregulated proteins are indicated in turquoise and upregulated proteins in red. The left panel of the graph shows the matrix that indicates intersects of significantly altered proteins between comparisons of mock-infected and SARS-CoV-2-infected cells using horizontal line bars.

Previous proteomics-based studies have observed that SARS-CoV-2 causes global proteomic changes after 48hpi specifically in pathways related to ErbB, HIF-1, mTOR, and TNF signaling (Appelberg et al., 2020); complement system and coagulation cascades (Tiwari et al., 2020); and interferon signaling (Chen et al., 2021). In order to understand the delayed changes in Huh7 cells (Huh7^48h^) compared with Calu-3 and Caco2 cells, we extracted the proteomics dataset from our earlier study (Appelberg et al., 2020). Next, we aligned the significantly changed proteins in this dataset with that of the Calu-3 and Caco2 cells. There were 42 common proteins that were dysregulated in all three cell lines (Figure 6A), showing a similar trend in 15 proteins (8 were commonly upregulated and 7 were commonly downregulated) and opposite trends in 27 proteins in all the three cell lines (Figure S8). We employed Reactome pathway analysis to define the commonly dysregulated pathways using 42 proteins and observed that pathways related to interferon signaling were the top hits (Figure 6B). We looked in detail into the 24 proteins that belonged to pathways related to IFN-signaling and were significantly altered in Calu-3 and Caco2. As shown in Figure 6C, only STAT2, STAT1, DDX58, ISG15, and IFIT1 were commonly upregulated in all three cell lines. Distinct features were noticed in B2M, PML, HLA-E, and HLA-B in Calu-3 (downregulated), Caco2 (upregulated), and Huh7^48h^ (no change). IFI35 was upregulated in both Calu-3 and Caco2 cells but was downregulated in Huh7^48h^.

### Cell-type- and virus-specific differences in proteome

There are obvious commonalities and distinct features between different cell lines that could be defined by the tissue of origin. Till now we have shown that SARS-CoV-2 commonly regulates the type-I IFN signaling in cell lines derived from different tissues. In order to determine which other pathways are distinctively regulated by SARS-CoV-2 in Calu-3, Caco2, and Huh7^48h^ cells, we identified the proteins that were significantly uniquely altered by infection in each of the three cell lines. There were 3,897 proteins in Calu-3, 1,347 proteins in Huh7^48h^, and 38 proteins in Caco2 that uniquely changed (Figure 6A). A Reactome pathway analysis on these proteins revealed major changes in pathways related to mitochondrial processes in Calu-3 (Figure S9A) and eukaryotic translation processes in Huh7^48h^ (Figure S9B). We could not identify clearly any specific pathway modulated in Caco2. In addition, we investigated altered pathways shared between only Huh7^48h^ and Caco2 and only Huh7^48h^ and Calu-3. As presented in Figures S10A and S10B respectively, unlike Caco2 and Calu-3 they did not show shared characteristics of IFN-signaling dysregulation as top pathways.

## Discussion

Cellular models that reproduce the SARS-CoV-2 life cycle are essential to understand the viral-host interplay and to test new antivirals. Vero-E6 cell line, which originates from African green monkey kidney cells, is often a choice for cell-culture-based infection model for coronavirus research (Ogando et al., 2020), because it expresses the receptor ACE2 and shows prominent cytopathogenicity with efficient virus production. However, Vero-E6 may not be a suitable cell model to study the pathophysiology of the cell in response to the virus infection, as it lacks genes encoding type-I interferons (Osada et al., 2014). Cell lines of human origin are more relevant. In the present study, we systematically analyzed SARS-CoV-2 susceptibility and cytopathogenicity in cell lines originating from human lung (Calu-3, A549, 16HBE), colon (Caco2), liver (Huh7), and kidney (293FT). Furthermore, using proteomics we characterized the cellular changes caused by SARS-CoV-2 in the susceptible cell lines Calu-3, Caco2, 293FT, and Huh7. Our data provide insight into the cell-type-specific cellular re-organization caused by SARS-CoV-2.

**Figure 7:**
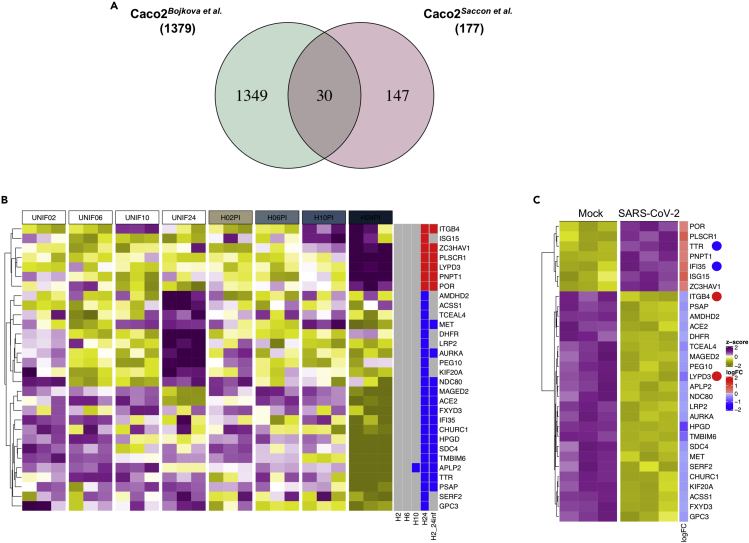
Validation of proteins dysregulated in Caco2 cell lines by SARS-CoV-2 infection from independent studies (A) Venn diagram and overlap of proteins with significant abundance between mock-infected and SARS-CoV-2-infected Caco2 cell lines at 24hpi from this study and a study by Bojkova et al. (B) Heatmap of significant overlapping proteins in mock-infected and SARS-CoV-2-infected Caco2 cells at 24 h in Bojkova et al. Data were quantile normalized and *Z* score transformed. Lower values are represented in yellow and higher values in purple. Significant differentially expressed proteins between time points are indicated in blue if downregulated and in red if upregulated. (C) Heatmap of the significant differentially abundant proteins of the Bojkova et al. in our dataset (Bojkova et al., 2020). Proteins following the opposite trend of expression in both the datasets are indicated by dots.

Several recent studies testing different local strains show conflicting results concerning susceptibility and cytopathogenicity in human cell lines (Figure 2C), mostly when Caco2 and Calu-3 cells are employed. The Swedish isolate tested here showed high virus production and prominent cytopathogenicity in Calu-3 cells. However, it needs to be pointed out that the susceptibility of the Calu-3 cells to SARS-CoV-2 increased following 72 h of incubation before infection, as with 24 h of incubation of the cells prior to infection showed a very moderate virus production and a delayed CPE (Figure 1). Polarized Calu-3 cells were previously reported to enhance SARS-CoV infection (Tseng et al., 2005). We observed morphological changes in Calu-3 cell monolayer after 72 h of incubation with a distinct distribution of β-catenin bordering the cells (Figure S1) tempting us to speculate polarization in these cells that lead to enhanced susceptibility. Another observation with Calu-3 cells was that upon infection, they did not show a CPE similar to Vero-E6; rather the Calu-3 stopped growing, rounded up, and mottled, as compared with the uninfected control. The morphological change observed in Calu-3 might represent a cellular mechanism to control viral infection and therefore requires more investigation. Several other studies did not align with our findings, as the strain from Munich (Zecha et al., 2020) and Japan (Matsuyama et al., 2020) showed very poor susceptibility and the Hong Kong strain showed a high susceptibility with no cytopathogenicity (Chu et al., 2020; Hui et al., 2020). The discrepant susceptibility could be dependent on the polarization of the Calu-3 cells where ACE2, the receptor for SARS-CoV-2, is expressed apically in polarized Calu-3 cells and has been shown to facilitate entry and release of the SARS-CoV (Tseng et al., 2005). Caco2 was another cell line that showed a varied level of susceptibility in different studies. In Caco2 although we observed high viral susceptibility, we did not observe any appreciable cytopathogenicity as was reported for the French (Wurtz et al., 2021) and the Honk Kong strain (Chu et al., 2020). Bojkova et al. have performed a time-course proteomic study with the Frankfurt strain of SARS-CoV-2 in infected Caco2 cells over a period of 24 h (Bojkova et al., 2020). Contrary to our results (Figure 1), the authors observed cytopathogenicity in Caco2 cells at 24hpi. In order to compare their 24hpi proteomic data with ours we re-analyzed their data with similar statistical considerations as ours. Compared with ours, they observed a very high number of proteins to be significantly altered (1,379 versus 177), among which we observed only 30 proteins overlapping in both the studies (Figure 7A). A heatmap of the 30 common proteins over time in the Bojkova et al. data is shown in Figure 7B, and the heatmap of the same proteins in our study at 24hpi is shown in Figure 7C. We observed discordance in four proteins, where TTR and IFI35 were upregulated in their study but downregulated in ours, and ITGB4 and LYPD3 were downregulated in their study but were upregulated in ours. We also specifically looked into the proteins related to IFN-signaling pathways and observed several nuclear transporters to be upregulated in the Bojkova et al. study. Among the ISGs, only ISG15 showed an upregulation in both the data (Figure S11). Of note, unlike others, the Frankfurt strain was the only strain that was isolated and adapted in Caco2 that could have possibly led to higher susceptibility and CPE of this strain in the cell line (Bojkova et al., 2020). In addition, different infective doses, structural changes in the virus, culture conditions, and clonal differences in the cell lines could also govern strain-specific differences in the cellular tropism of SARS-CoV-2. Global proteomics analysis upon SARS-CoV-2 infection was also performed by us in Huh7 cells and by others in Vero-6 cells (Grenga et al., 2020; Zecha et al., 2020) and A549-ACE2-expressing cells (Stukalov et al., 2020). All these proteomics studies have pointed toward dysregulation majorly in the Akt-mTOR signaling (Appelberg et al., 2020; Bojkova et al., 2020; Stukalov et al., 2020), components of spliceosome and RNA modification (Appelberg et al., 2020; Bojkova et al., 2020; Stukalov et al., 2020; Zecha et al., 2020), cell adhesion pathways (Zecha et al., 2020), and pathways linked to metabolism including central carbon metabolism (Bojkova et al., 2020; Grenga et al., 2020; Stukalov et al., 2020; Zecha et al., 2020). Of note, we have also analyzed our data from the four cell lines targeting central carbon metabolic pathways and observed that only Calu-3 cells showed changes in protein associated to the TCA cycle, glycolysis/gluconeogenesis, and fructose and mannose metabolism (Krishnan et al., 2021).

A major role in susceptibility to viral infection is played by the interaction between viral envelope glycoproteins and cellular receptors, which initiate the early phases of the viral life cycle. Therefore, we first investigated whether mutations in the spike glycoproteins of the different strains presented above could affect their tropism and cytopathogenicity. However, no major amino acid changes were observed in the spike protein, except for the Munich strain harboring D614G mutation that had become the dominant genotype in Europe and considered to be more infectious in humans (Korber et al., 2020). Interestingly, both Caco2 and Calu-3 were not susceptible to the Munich strain in the study performed by Zecha et al., and this correlated with the absence of ACE2 expression in their steady-state proteomics data obtained from Caco2 and Calu-3 cell lines (Zecha et al., 2020). ACE2 and TMPRSS2 proteins are considered essential for SARS-CoV-2 entry, and ACE2 expression alone strongly correlated with the virus susceptibility. We observed a strong correlation between ACE2 expression and virus tropism, whereas increased expression of TMPRSS2 alone in Huh7 and 293FT cell lines showed susceptibility to SARS-CoV-2, perhaps due to TMPRSS2-mediated enhanced virus uptake into the cell (Heurich et al., 2014). However, we cannot exclude the possibility that other molecules or endocytosis mechanisms are involved in viral recognition and entry.

In addition, in the present study, we have investigated the replicative capacity of the virus by measuring the presence of newly produced virions in the culture supernatant. One interesting observation was that we did not observe any virus budding in the cell surface of Huh7 and 293FT, in spite of increased viral RNA in the supernatant over time, differently from Caco2 and Calu-3 (Figure 2). Recently, it has been shown that beta-coronaviruses can hijack the lysosomal organelles resulting in non-lytic exocytosis of the virus (Ghosh et al., 2020). Overall, our data on SARS-CoV-2 virus production, release, receptor expression, and cytotoxicity highlight the cell-line-specific SARS-CoV-2 life cycle and suggest different mechanisms of remodeling of the host cellular environment during infection, which we addressed by quantitative proteomics. Although there were definite virus-specific proteome changes (Figure S9), the pathways that were commonly regulated between Caco2 and Calu-3 belonged to type-I interferon signaling. It was also evident that the dynamics of the viral life cycle influences the proteomic landscape or vice versa. As in Caco2 and Calu-3 an efficient production of the virus correlated with increased activation of RIG-I/MDA5 signaling and subsequently IFN-β and ISGs by 24hpi (Figures 4 and 5). However, this was not observed in 293FT and Huh7. In another study by us, we have observed that in Huh7 cells SARS-CoV-2 can inhibit type-I IFN signaling early, and the dysregulation in this pathway is only observed at 48hpi (Chen et al., 2021), which correlates with moderate production of the virus. In spite of differences in the dynamics of the cellular response, type-I IFN pre-sensitization of the Caco2, Calu-3 (Shuai et al., 2020), and Huh7 (Chen et al., 2021) has been shown to effectively inhibit SARS-CoV-2 infection. In addition, many of the ISGs such as IFIT1, ISG15, and DDX58 were upregulated in all three cell lines (Figure 6). Induction of type I and type II IFN that was proportional to the viral load was also noted in a proteomics study performed in autopsy lung material of fatal COVID-19 cases and lung tissues obtained from SARS-CoV-2-infected non-human primates in a longitudinal manner (Kalocsay et al., 2020). Another recent study performed on autopsy tissues from various organs observed that COVID-19 patients with coronary heart disease had upregulation of multiple protein belonging to RIG-I signaling pathway, and overall, interferon gamma receptor 1 (IFNGR1) was dysregulated in all the major organs except for the thyroid and testes (Nie et al., 2021). These findings suggest that even in the presence of cell-type-specific diversity in cellular responses, there are common pathways that could be efficiently targeted to inhibit SARS-CoV-2. This is particularly relevant because SARS-CoV-2 can infect different organs of the body (Mallapaty, 2020; Trypsteen et al., 2020).

In conclusion, we identify some cell lines of human origin that could be used to study the biological properties of SARS-CoV-2. In addition, we observed that type-I interferon is commonly regulated during infection in cell lines originating from lungs, colon, and liver and thus deserving more mechanistic studies to identify factors that could be utilized to control the infection.

### Limitations of the study

We acknowledge some limitations of our study. Foremost is that the analysis is restricted to cell lines, which may not be physiologically representative of the human tissue, like other *ex vivo* systems such as organoids. However, the use of cell lines can still provide an overview of the complexity and variability of the interaction between SARS-CoV-2 and the human cellular targets. Furthermore, we restricted our proteomics study to 24hpi, and more detailed time kinetics experiments are required to elucidate better the dynamic changes occurring during infection. Because Calu-3 and Caco2 are more susceptible to SARS-CoV-2 than Huh7 and 293FT, the infection kinetics will be very different to compare at a particular time point. However, we tried to apprehend a time point where the virus production is at its exponential phase before reaching a plateau and without causing any cytopathogenicity (Figure 1). In addition, even though we tried to compile and compare the infectivity and cytotoxicity of SARS-CoV-2 in model cell lines reported in different studies, variability is still occurring from lab to lab, specifically concerning culturing techniques, cell strains or passages, infective dose, and many others.

### Resource availability

#### Lead contact

Further information and requests for resources and reagents should be directed to and will be fulfilled by the lead contact, Soham Gupta, soham.gupta@ki.se.

#### Materials availability

No new reagents were created in this study.

#### Data and code availability

The mass spectrometry proteomics data (raw MS files and search files) have been deposited to the ProteomeXchange Consortium (http://proteomecentral.proteomexchange.org) via the PRIDE partner repository with the accession number PXD023760. All the generated codes are deposited in GitHub (https://github.com/neogilab/COVID_cell_lines). All the unprocessed and processed proteomics raw data are uploaded as supplementary tables (Tables S4, S5, S6, S7, S8, S9, S10, and S11). Additional supplemental information are available from Mendeley Data at. https://doi.org/10.17632/tjr7cfhwm7.1. Information regarding any additional data is available from the corresponding author on reasonable request.

## Methods

All methods can be found in the accompanying transparent methods supplemental file.

## Supplemental information

Document S1. Transparent methods, Figures S1–S13, and Tables S1–S3

Table S4. 293FT and Huh7 TMTpro raw peptide groups abundances, related to figure 3 Peptide list containing raw TMTpro-labeling abundances across the samples. Peptide identifications were obtained from 12 HpH-RPLC fractions. All entries were considered with 1% FDR.

Table S5. 293FT and Huh7 TMTpro raw protein groups abundances, related to figure 3 Master protein list calculated from peptide identifications. Each entry contains raw abundances for quantification across the samples. All entries were considered with 1% FDR.

Table S6. Calu-3 and Caco2 TMTpro raw peptide groups abundances, related to figure 3 Peptide list containing raw TMTpro-labeling abundances across the samples. Peptide identifications were obtained from 12 HpH-RPLC fractions. All entries were considered with 1% FDR.

Table S7. Calu-3 and Caco2 TMTpro raw protein groups abundances, related to figure 3 Master proteins list calculated from peptide identifications. Each entry contains raw abundances for quantification across the samples. All entries were considered with 1% FDR.

Table S8. 293FT normalized protein abundances: protein list of quantile normalized abundances for 293FT cells, related to figure 3

Table S9. Huh7 normalized protein abundances: protein list of quantile normalized abundances for Huh7 cells, related to figure 3

Table S10. Calu-3 normalized protein abundances: protein list of quantile normalized abundances for Calu2 cells, related to figure 3

Table S11. Caco2 normalized protein abundances: protein list of quantile normalized abundances for Caco3 cells, related to figure 3

## Footnotes

Supplemental information can be found online at https://doi.org/10.1016/j.isci.2021.102420.
