# Supplementary material for: Cell-type-resolved quantitative proteomics map of interferon response against SARS-CoV-2
Source: iScience. 2021 Apr 20;24(5):102420. doi: 10.1016/j.isci.2021.102420 (PMC8056843; doi:10.1016/j.isci.2021.102420)
Supplement: Document S1. Transparent methods, Figures S1–S13, and Tables S1–S3 [file mmc1.pdf]

iScience, Volume *24*

Supplemental information

Cell-type-resolved quantitative proteomics map of interferon response against SARS-CoV-2

Elisa Saccon, Xi Chen, Flora Mikaeloff, Jimmy Esneider Rodriguez, Laszlo Szekely, Beatriz Sá Vinhas, Shuba Krishnan, Siddappa N. Byrareddy, Teresa Frisan, Ákos Végvári, Ali Mirazimi, Ujjwal Neogi, and Soham Gupta

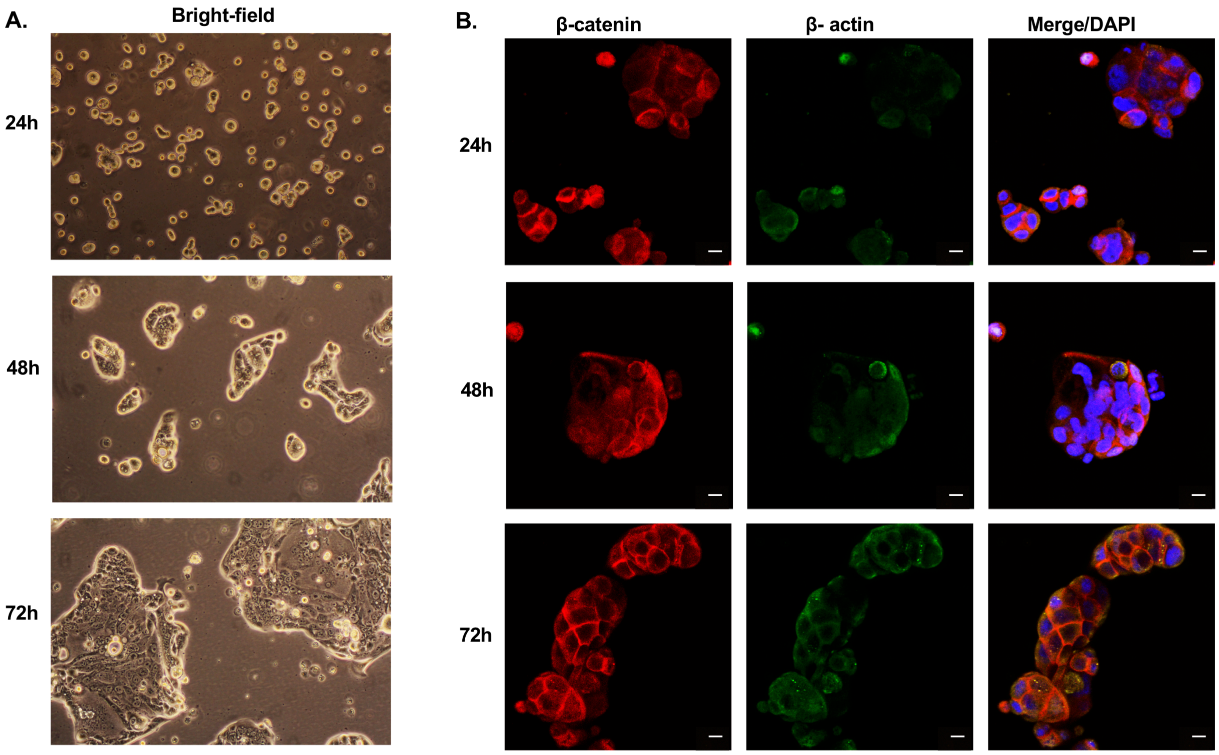

Figure S1: Morphological changes in Calu-3 cells with longer incubation time, related to Figure 1. **A.** Bright-field image of Calu-3 cells on a cell culture plate at 24h, 48h and 72h after seeding showing formation of clusters of cells with defined border. Images were captured using Canon EOS60D with microscope adapter mounted on Axiovert 40C inverted microscope (Zeiss) under 10x objective. Scale bar is not available. **B.** Confocal microscopy images of Calu-3 cells at 24h, 48h and 72h after seeding stained with β-actin (Green), β-catenin (Red) and nuclei (DAPI, blue) obtained with 20x objective. The observed fluorescent intensity of β-actin increased over time showing defined borders around the cells. The white scale bars in each panel denotes 10μM.

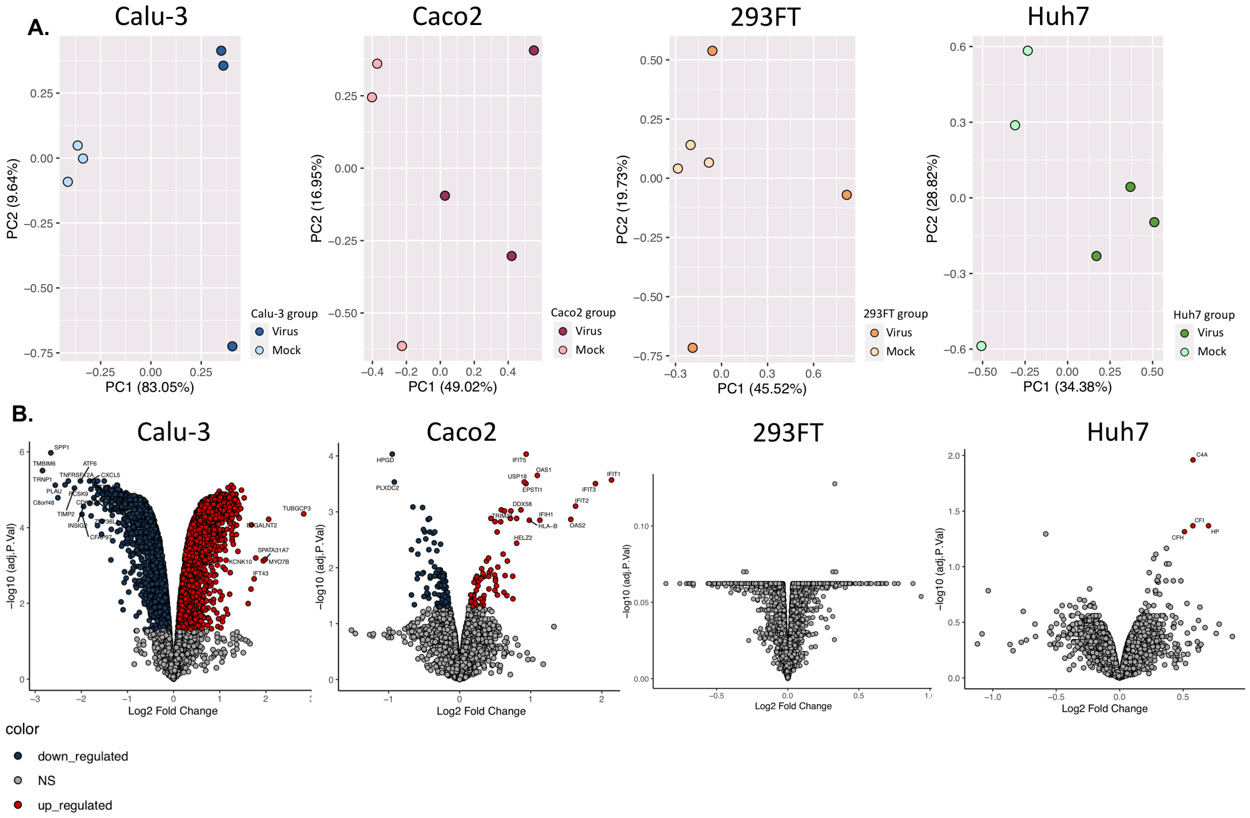

Figure S2: Quantitative proteomics of SARS-CoV-2 infected cell lines, related to Figure 3. **A.** PCA plot of the quantitative proteomics of the SARS-CoV-2-infected (Virus) and Mock-infected (Mock) Calu-3, Caco2, 293FT and Huh7 cell lines. R1, R2 and R3 represents technical triplicates. **B.** Volcano-plot of proteomics data in SARS-CoV-2-infected Calu-3, Caco2, 293FT and Huh7 cell lines compared to the Mock-infected cells depicted as adjusted p-values vs. Fold change. Red dots show significantly upregulated proteins and blue dots show significantly downregulated proteins. Top 20 significantly altered proteins are indicated in the graph.

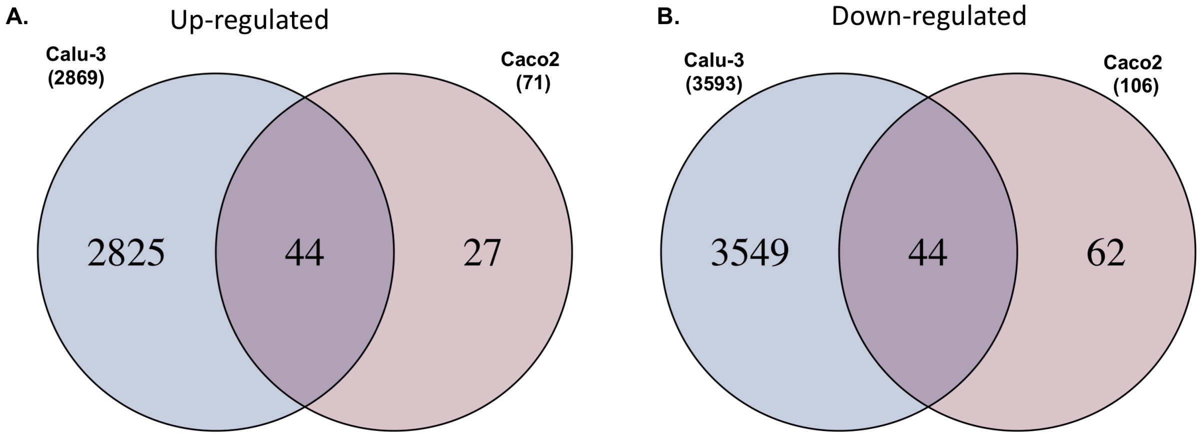

Figure S3: Overlap of differential protein abundance in SARS-CoV-2-infected Calu-3 and Caco2 cells, related to Figure 4. **A.** Venn diagram of proteins with higher abundance in Calu-3 and Caco2 cell lines after SARS-CoV-2 infection compared to mock infection. **B.** Venn diagram of proteins with lower abundance in Calu-3 and Caco2 cell lines after SARS-CoV-2 infection compared to mock infection.

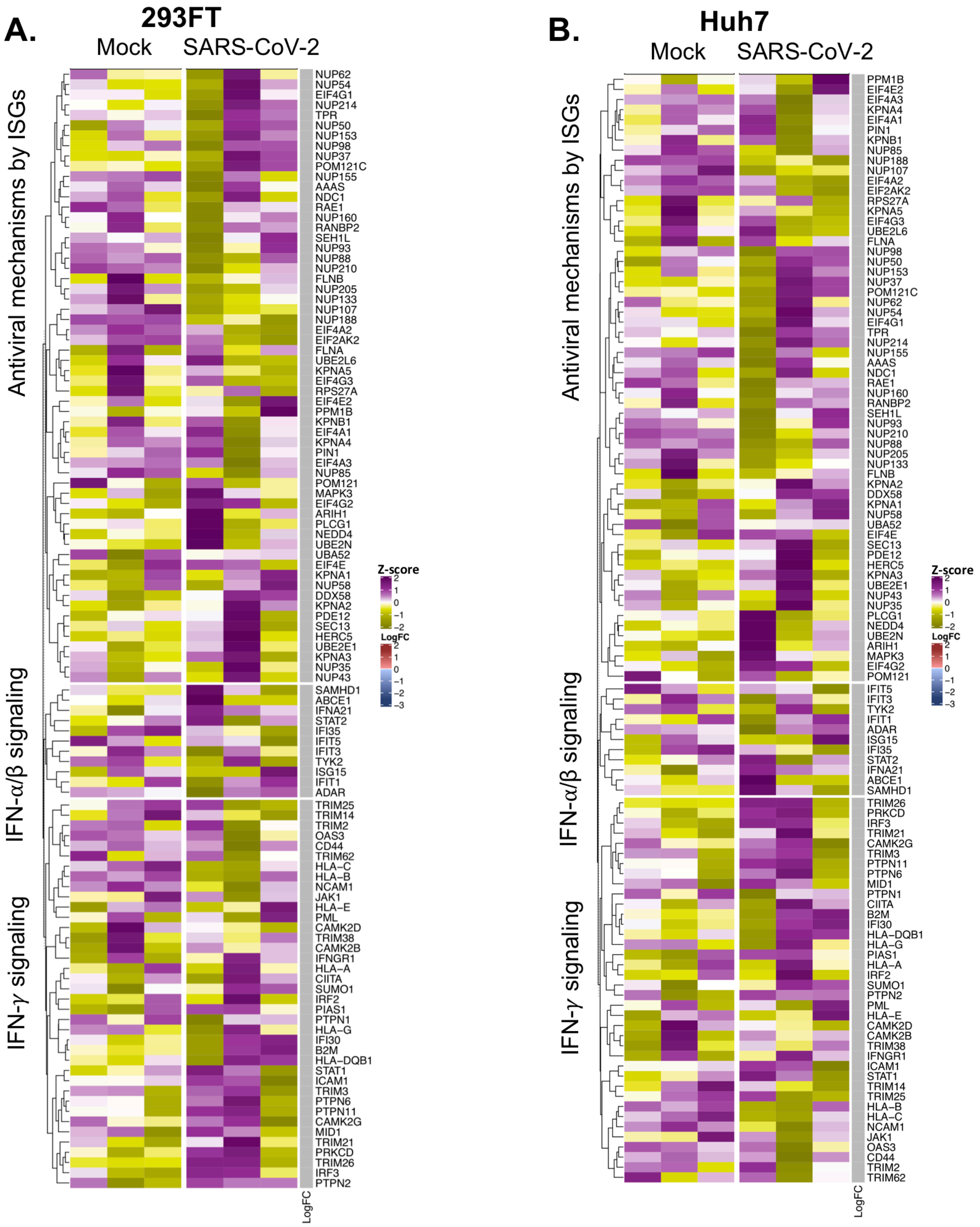

Figure S4: Interferon signaling pathways in Huh7 and 293FT after 24 hours of SARS-CoV-2 infection, related to Figure 4. Heatmaps representing the number of significant proteins (LIMMA, FDR < 0.05) between mock-infected and SARS-CoV-2-infected at 24hpi in **A.** 293FT and **B.** Huh7 cell lines.

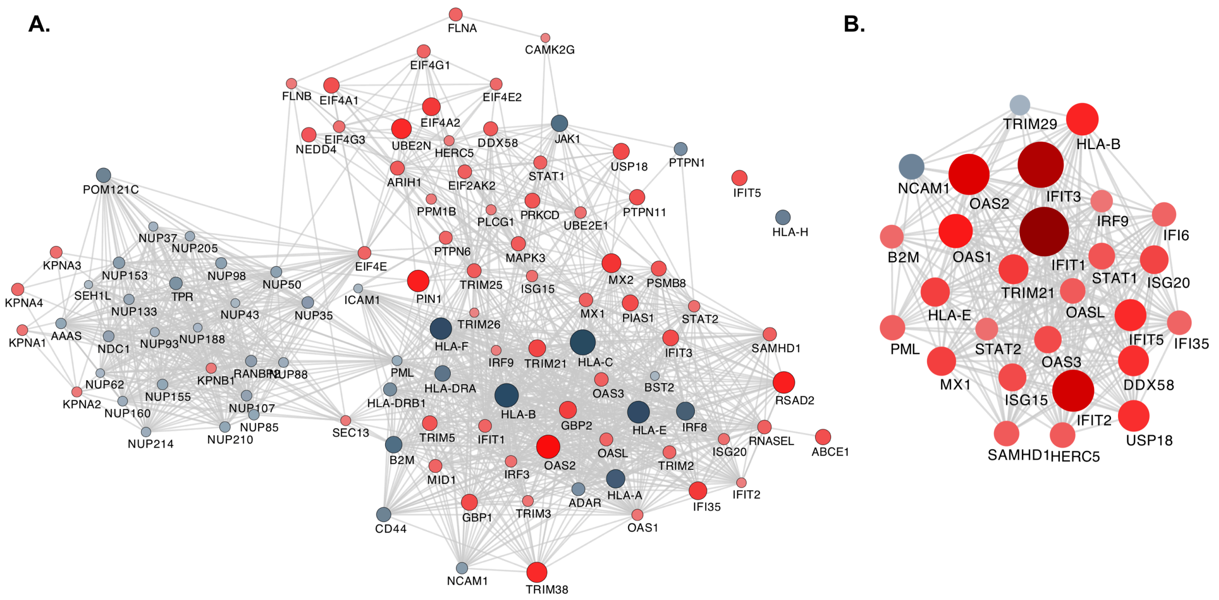

Figure S5. Network of differentially expressed proteins of interferon signaling pathways in SARS-CoV-2 infected Calu-3 and Caco2, related to Figure 4. Cytoscape networks of differentially abundant IFN-stimulated proteins in **A.** Calu-3 and **B.** Caco2 cell lines. Proteins are represented as circles. Gradient color was applied on proteins depending on fold change (low = blue to high = red). Size of protein is proportional to the fold change.

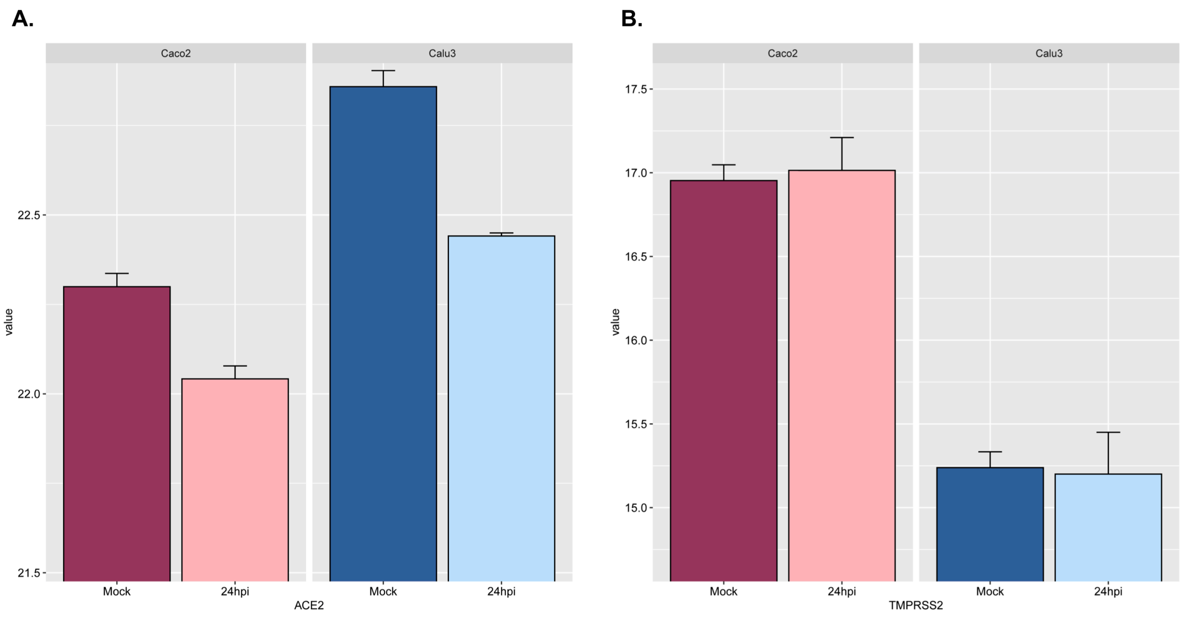

Figure S6. Expression of SARS-CoV-2 entry receptors in Calu-3 and Caco2 upon infection with SARS-CoV-2, related to Figure 4. Expression levels of **A**. ACE2 receptor and **B**. TMPRSS2 receptor in Caco2 and Calu-3 cells upon SARS-CoV-2 infection at 24hpi as quantified by TMT-labeling based proteomics.

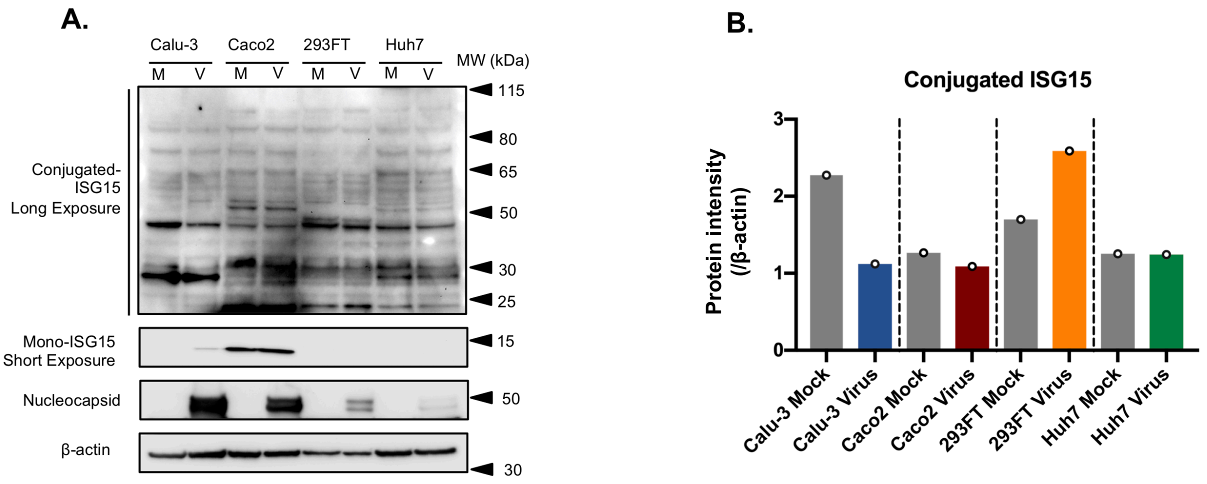

Figure S7. ISG15 expression and ISGylation of host cellular proteins in SARS-CoV-2-infected Calu-3, Caco2, 293FT and Huh7 cell lines, related to Figure 5. **A**. ISG15 protein levels in SARS-CoV-2-infected or mock-infected cells. The representative western blots with indicated antibodies are shown. **B**. The intensity of the conjugated ISG15 bands were quantified by ImageJ and protein intensity compared to β-actin is shown.

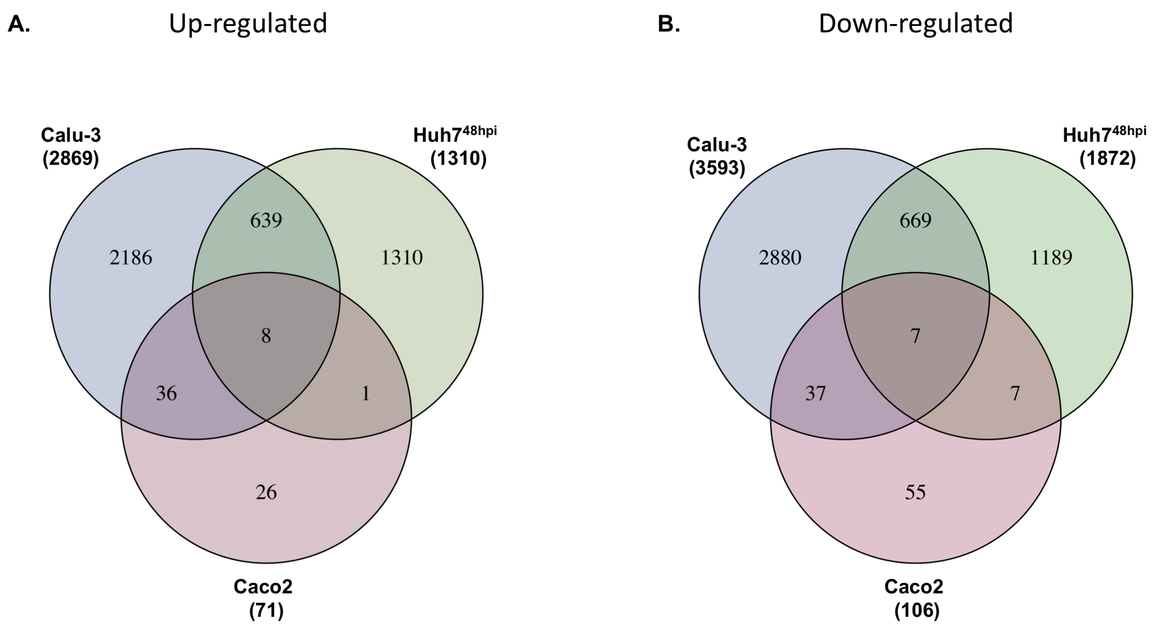

Figure S8. Overlap of Differential protein abundance in SARS-CoV-2-infected Calu-3, Caco2 and Huh7 cells, related to Figure 6. **A**. Venn diagram of proteins with higher abundance in Caco2 and Calu-3 cells after 24h of SARS-CoV-2 infection and Huh7 cell lines after 48h of SARS-CoV-2 infection compared to mock infection. **B**. Venn diagram of proteins with lower abundance in Caco2 and Calu-3 cells after 24h of SARS-CoV-2 infection and Huh7 cell lines after 48h of SARS-CoV-2 infection compared to mock infection.

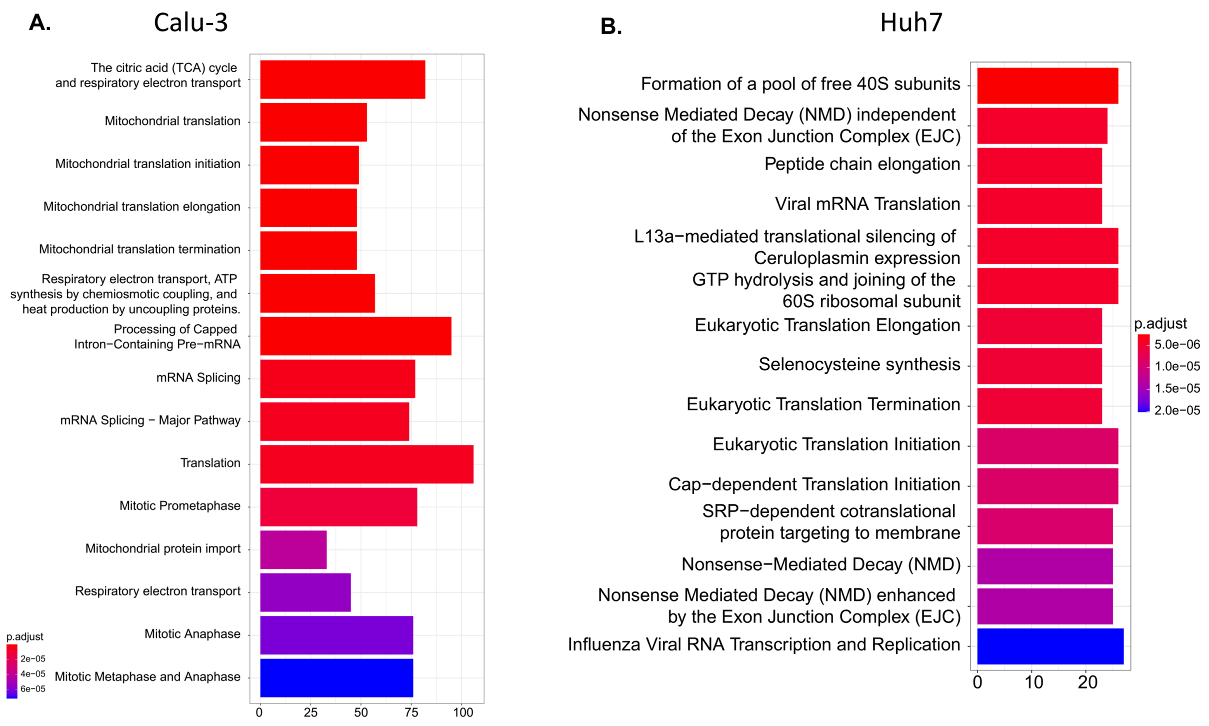

Figure S9. Pathways uniquely enriched in Calu-3 and Huh7 cells, related to figure 6. **A.** ReactomePA barplot enrichment map of significant proteins identified in Calu-3 cell line excluding Caco2 and Huh7 cell lines. **B.** ReactomePA barplot enrichment map of significant proteins identified in Huh7 cell line excluding Caco2 and Calu-3 cell lines.

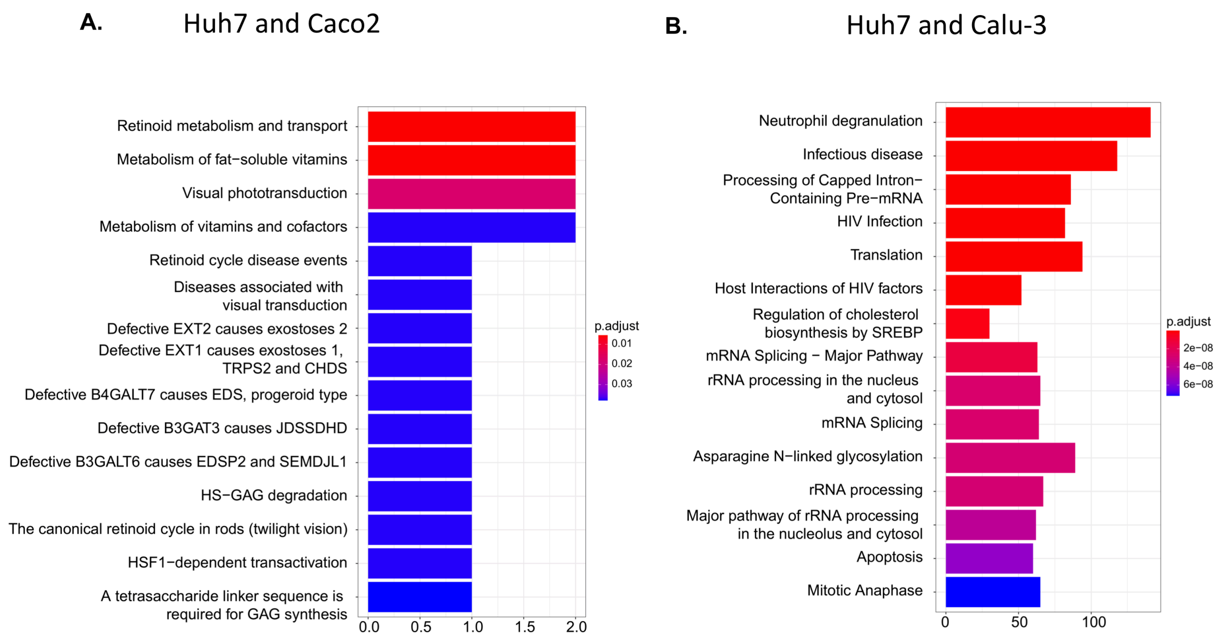

Figure S10. Pathways commonly enriched in any two cell lines, related to figure 6. **A.** ReactomePA barplot enrichment map of significant overlapping proteins in Huh7 and Caco2 cell lines. **B.** ReactomePA barplot enrichment map of significant overlapping proteins in Huh7 and Calu-3 cell lines.

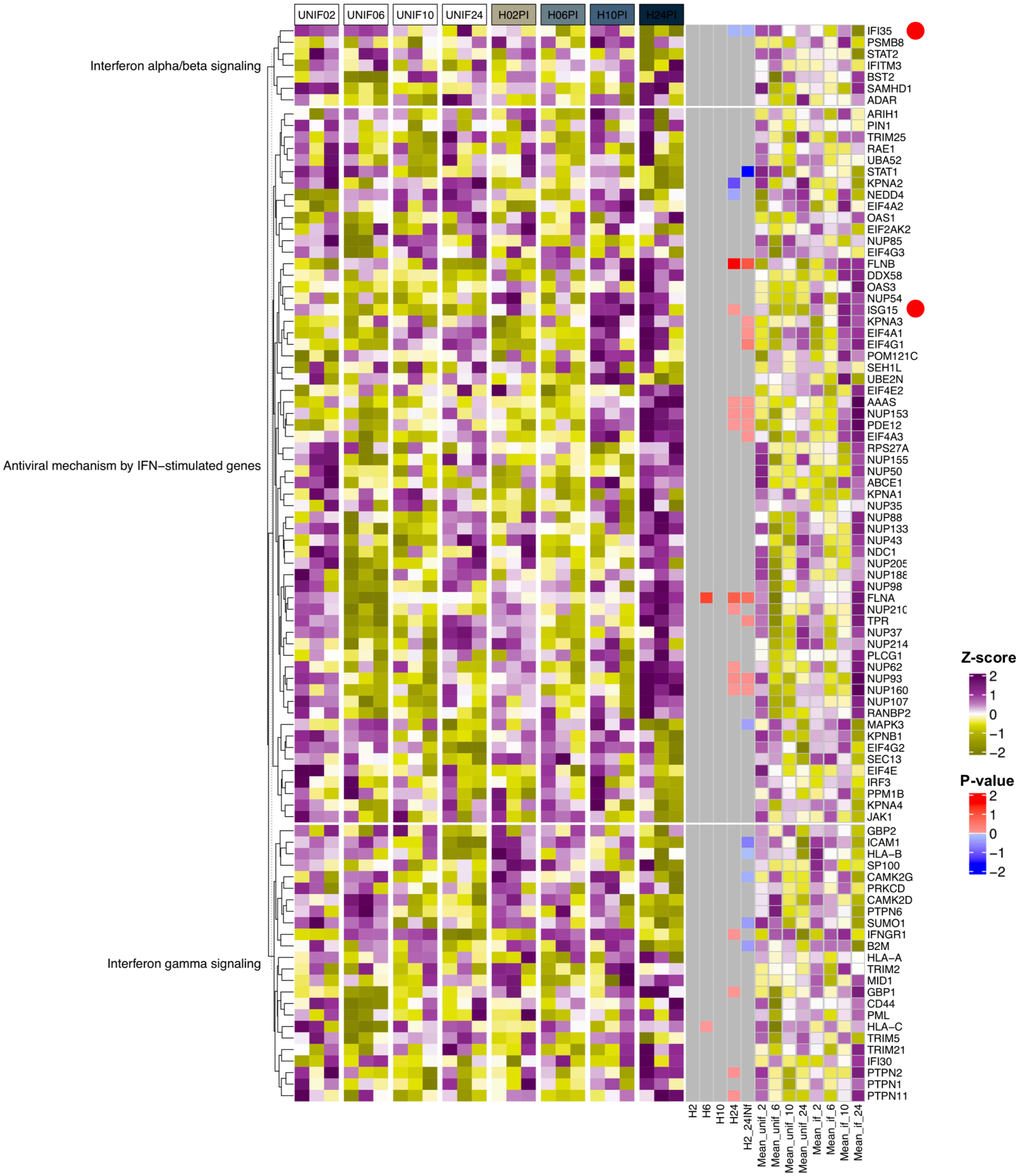

Figure S11. Re-analysis of data from Bojkova *et al.* paper, related to Figure 7. Heatmap representing the number of significant proteins (LIMMA, FDR < 0.05) belonging to interferon-signaling pathways between mock-infected and SARS-CoV-2-infected Caco2 cell line at different time points. Red dots indicate proteins that were significantly upregulated in our experiment.

Table S1: Primers and probes used for RT-qPCR of viral RNA, related to Figure 1 and Figure 3.

Name	SARS-CoV-2 E gene
Forward Sequence (5' to 3')	ACAGGTACGTTAATAGTTAATAGCGT
Reverse Sequence (5' to 3')	ATATTGCAGCAGTACGCACACA
Probe Sequence (5' to 3')	[FAM] ACACTAGCCATCCTTACTGCGCTTCG [BBQ650]
**Name**	**SARS-CoV-2 N gene**
Forward Sequence (5' to 3')	CACATTGGCACCCGCAATC
Reverse Sequence (5' to 3')	GAGGAACGAGAAGAGGCTTG
Probe Sequence (5' to 3')	[FAM] ACTTCCTCAAGGAACAACATTGCCA [BBQ650]

Table S2: List of references showing cell line susceptibility to SARS-CoV-2, related to Figure 2.

Place of origin (Reference)	Strain(s)	MOI	Method	Criteria	
Sweden *(Saccon et al).*	SWE/01/2020	1 and 0.1	Viral N-gene RT-qPCR from cell culture supernatant	High	>3 fold increase in log_{10} RNA copies by 48hpi
				Modest	1 fold increase in log_{10} RNA copies by 48hpi
				Low	No difference in log_{10} RNA copies by 48hpi
Frankfurt, Germany *(Bojkova et al., 2020)*	FFM1/2020	0.01	Viral RNA RT-qPCR from cell culture supernatant	High	10^5 fold increase in virus genome/mL by 48hpi
				Low	10 fold increase in virus genome/mL by 48hpi
Munich, Germany *(Zecha et al., 2020)*	Mun-IMB1/2020	3	Fluorescence from GFP-SARS-CoV-2reporter	Low	No difference in GFP fluorescence by 24hpi
France *(Wurtz et al., 2020)*	IHUMI2	0.1	Viral E-gene RT-qPCR from cell culture supernatant	High	Around 6 ΔCt between day 0 and day 7
USA *(Jureka et al., 2020)*	WA1/2020	0.01	Supernatant viral titer quantified by plaque assay	High	10^7 pfu/mL by 48hpi
				Modest	10^2-10^3 pfu/mL by 48hpi
				No	<10 pfu/mL by 48hpi
Canada *(Banerjee et al., 2020)*	SB3-TYAGNC	0.01	Supernatant viral titer quantified using Spearman and Karber's method	High	0 $TCID_{50}$/mL by 24hpi to $2x10^4$ by 48hpi and $2x10^6$ at 72hpi
China *(Liao et al., 2020)*	KMS1/2020	0.2	Viral genome copy number in the supernatant and cells by qRT-PCR + Virus infectious titres in supernatant by plaque assay	High	qRT-PCR: Supernatant – around 10^3 copies/mL increasing from 24 to 48hpi; Cells – around 10^5 copies/10^5 cells 24hpi increasing to 10^6 by 48hpi Titration: around 10^3 pfu/mL increasing from 24 to 48hpi
Hong Kong *(Chu et al., 2020)*	HKU-001a/2020 and VM20001061/2020	0.1	Viral genome RT-qPCR from cell culture supernatant	High	Significant difference in Virus genome copies/mL between 2 and 120hpi (>1 fold log_{10} change)
				No	No significant difference in Virus genome copies/mL between 2 and 120hpi
Taiwan *(Hsin et al., 2020)*	NTU01/TWN/2020 and NTU02/TWN/2020	0.1 (Huh7, A-549) and 0.01 (Huh7,A-549, Calu-3)	Viral RNA RT-qPCR from cell culture supernatant	High	Calu-3: 10 fold increase in vRNA copies/µg total RNA from 24 to 48hpi
				Modest	A-549 and Huh7: no significant increase in vRNA copies/µg total RNA from 24 to 48hpi at both MOIs.
Japan *(Matsuyama et al., 2020)*	TY-WK-521/2020	NA	Viral E and N-gene RT-qPCR from cell culture supernatant	Low	E-gene: Cq value of around 31-32 at 48hpi; N-gene: Cq value of around 33-34 at 48hpi

Table S3: qPCR primers used to measure ISG expression, related to Figure 5.

**Name**	**Forward sequence (5' to 3')**	**Reverse sequence (5' to 3')**
IFN-β	TCCAAATTGCTCTCCTGTTG	GCAGTATTCAAGCCTCCCAT
IFIT1	TCTCAGAGGAGCCTGGCTAA	TGACATCTCAATTGCTCCAGA
MX1	CCAGCTGCTGCATCCCACCC	AGGGGCGCACCTTCTCCTCA
MX2	CAGAGGCAGCGGAATCGTAA	TGAAGCTCTAGCTCGGTGTTC
ISG15	CGCAGATCACCCAGAAGATCG	TTCGTCGCATTTGTCCACCA
RIG-I	ATCCCAGTGTATGAACAGCAG	GCCTGTAACTCTATACCCATGTC
GAPDH	TGGGCTACACTGAGCACCAG	GGGTGTCGCTGTTGAAGTCA

Table S4: 293FT and Huh7 TMTpro Raw Peptide groups abundances, related to Figure 3. Peptide list containing raw TMTpro-labeling abundances across the samples. Peptide identifications were obtained from 12 HpH-RPLC fractions. All entries were considered with 1% FDR.
(Provided as Excel Table)

Table S5: 293FT and Huh7 TMTpro Raw protein groups abundances, related to Figure 3. Master protein list calculated from peptide identifications. Each entry contains raw abundances for quantification across the samples. All entries were considered with 1% FDR.
(Provided as Excel Table)

Table S6: Calu-3 and Caco2 TMTpro Raw Peptide groups abundances, related to Figure 3. Peptide list containing raw TMTpro-labeling abundances across the samples. Peptide identifications were obtained from 12 HpH-RPLC fractions. All entries were considered with 1% FDR.
(Provided as Excel Table)

Table S7: Calu-3 and Caco2 TMTpro Raw protein groups abundances, related to Figure 3. Master proteins list calculated from peptide identifications. Each entry contains raw abundances for quantification across the samples. All entries were considered with 1% FDR.
(Provided as Excel Table)

Table S8: 293FT normalized protein abundances: Protein list of quantile normalized abundances for 293FT cells, related to Figure 3.
(Provided as Excel Table)

Table S9: Huh7 normalized protein abundances: Protein list of quantile normalized abundances for Huh7 cells, related to Figure 3.
(Provided as Excel Table)

Table S10: Calu-3 normalized protein abundances: Protein list of quantile normalized abundances for Calu2 cells, related to Figure 3.
(Provided as Excel Table)

Table S11: Caco2 normalized protein abundances: Protein list of quantile normalized abundances for Caco3 cells, related to Figure 3.
(Provided as Excel Table)

Table S12: Differential protein abundance (DPA) global in SARS-CoV-2-infected cells compared to mock-infected cells, related to Figure 3, Figure 6 and Figure 7.

Cell line	Detected	DPA
293FT	8977	0
Huh7	7623	4
Caco2	8784	177
Calu-3	8768	6462
Caco2 (Bojkova et al., 2020)	6258	1448 (24h)
Huh7 (Appelberg et al., 2020)	8991	3830

Table S13: Differential protein abundance (DPA) of interferon-regulated genes in SARS-CoV-2-infected cells compared to mock-infected cells, related to Figure 4, Figure 6 and Figure 7.

Cell line	Detected (IFN)	DPA (IFN)
293FT	109	0
Huh7	109	0
Caco2	131	27
Calu-3	129	105
Caco2 (Bojkova et al., 2020)	93	17 (24h)
Huh7 (Appelberg et al., 2020)	97	46

Transparent methods

Cells and viruses

The hepatocyte derived cellular carcinoma cell-line Huh7 was obtained from Marburg Virology Lab (Germany) matching the STR reference profile of Huh7 (Rohde et al., 2019), Vero-E6 (ATCC® CRL-1586™), Calu-3 (ATCC#HTB-55), A549 (ATCC#CCL-185) were obtained from ATCC (USA) and Caco2 cells was obtained from CLS cell line services, GmbH, Germany (#300137). 16HBE was obtained from Lena Palmberg, Karolinska Institutet (Sweden) and 293FT (Invitrogen, #10769564) was obtained from Matti Sällberg, Karolinska Institutet (Sweden). The SARS-CoV-2 virus (SWE/01/2020) used in this study was isolated from a nasopharyngeal sample of a patient at Public Health Agency (Sweden) and the virus was confirmed as SARS-CoV-2 by genome sequencing (Genbank accession number MT093571).

Virus propagation and infection

SARS-CoV-2 was propagated in Vero-E6 and titrated by measuring the tissue culture infectious dose (TCID50) in Vero-E6 cells. For virus susceptibility, the different cell lines were seeded at a concentration of 10,000 cells/well in a 96-well plate, 24h prior to infection. Calu-3 cells were incubated either for 24h or 72h before infection. Infection was performed by incubating the cells with 100µL of DMEM (Sigma-Aldrich, Sweden) supplemented with 5% heat-inactivated FBS (ThermoFisher, Sweden) containing SARS-CoV-2 either at moi 1 or 0.1 for 1h at 37°C. Then, the medium was removed and replenished with fresh medium. The cells were incubated for 120hpi.

Virus production and cytotoxicity

The virus production in cell supernatant and the virus mediated cytotoxicity were determined at 3h, 24h, 48h, 72h, 96h and 120h post infection. The cytotoxicity was determined by measuring the cellular ATP by using Viral ToxGlo assay (Promega, Sweden) as per manufacturers guidelines. The virus RNA in the supernatant was determined by qRT-PCR targeting either the N-gene or the E-gene using Takara PrimeDirect probe, qRT-PCR mix (Takara Bio Inc, Japan) using the primers (Eurofins, USA) and probes (Sigma-Aldrich, UK) reported in supplemental table S1. qRT-PCR was performed as previously reported (Corman et al., 2020), with cycling conditions: initial denaturation 90°C for 3 min, reverse transcription 60°C for 5 min, followed by 45 cycles of 95°C

for 5 sec, 58°C for 30 sec. Relative quantification of viral copies was done by comparing to the serially diluted stock virus. The cytotoxicity and virus production data for the Vero-E6, 16HBE and Huh7 at moi 1 for the time points 3hpi, 24hpi, 48hpi and 72hpi was extracted from our previously published data (Appelberg et al., 2020).

Analysis of Published data

Previously published papers on susceptibility of cell-lines to SARS-CoV-2 was searched in Google, Pubmed and bioRxiv. Papers representing a strain originating from different countries and studying maximum number of cell lines among various studies were included. The representative studies and the criteria of selecting the degree of infectivity in these studies are presented in supplemental table S2. Wherever cytopathogenecity was examined it was performed visually.

Quantitative RT-PCR

Messanger RNA (mRNA) expression of a few ISG transcripts and human GAPDH were measured by qRT-PCR. The sequences of the primers are listed in supplemental table S3. Total RNA was extracted using Direct-zol™ RNA miniprep (Zymo Research, USA) and RNA concentration was assessed using a spectrophotometer (NanoDrop UV Visible Spectrophotometer, Thermofisher, USA). Reverse transcription was performed using a high capacity reverse transcription kit (Applied Biosystems, USA) for 10 min at 25°C, followed by 37°C for 120 min and 85°C for 5 min. Quantitative RT-PCR assays were setup using the Power SYBR Green PCR Master Mix (Applied Biosystems, UK) using 250nM of primer pairs with cycling conditions: initial denaturation 95°C for 10 min, followed by 40 cycles of 95°C for 15 sec, 60°C for 1 min. Melting curves were run by incubating the reaction mixtures at 95°C for 15 sec, 60°C for 20 sec, 95°C for 15 sec, ramping from 60°C to 95°C in 1°C/sec. The values were normalized to endogenous GAPDH. Fold change was calculated as: Fold Change = $2^{-\Delta(\Delta Ct)}$ where ΔCt = Ct target—Ct housekeeping and Δ(ΔCT) = ΔCt infected - ΔCt mock-infected/untreated, according to the Minimum Information for Publication of Quantitative Real-Time PCR Experiments (MIQE) guidelines.

Antibodies and chemicals

Mouse anti-ACE2 E11, (SC390851, RRID:AB_420535, 1:1000), mouse anti-TMPRSS2 H4 (sc-515727, 1:1000) and mouse anti-ISG15 (sc-166755, RRID:AB_2126308, 1:1000) were purchased from Santa Cruz Biotechnology Ltd (USA). Mouse anti-β-tubulin clone TUB2.1, (T5201, RRID:AB_609915, 1:1000) and mouse anti-β-actin (A5441, RRID:AB_476744, 1:5000) were purchased from Sigma-Aldrich (USA). Rabbit anti-RIG-I clone D14G6 (#3743, RRID:AB_2269233, 1:1000), rabbit anti-MDA5 clone D74E4 (#5321, RRID:AB_10694490, 1:1000), rabbit anti-p-STAT1 (#9167, 1:1000) and rabbit anti-pIRF-3 clone 4D4G (#4947, RRID:AB_823547, 1:1000) were purchased from Cell-Signaling Technologies (USA). Rabbit SARS-CoV-2 nucleocapsid (#BSV-COV-AB-04, 1:1000) was from Bioserv (UK). Rabbit anti-β-actin (ab8227, RRID:AB_2305186, IF: 1:100;) was purchased from Abcam (Cambridge, MA, USA). Mouse anti-β-catenin clone 17C2 (PA0088, RRID:AB_10555989, IF: 1:100) was purchased from Leica biosystems (Buffalo Grove, IL, USA). Alexa Fluor 488- and 568-conjugated secondary antibodies (1:1000) were from Thermo Fisher (A32731, RRID:AB_2633280 and A11004, RRID:AB_2534072, respectively).

DL-Dithiothreitol (DTT, D0632) was purchased from Sigma-Aldrich (USA). Complete protease inhibitor cocktail and phosphatase inhibitor cocktail were purchased from Roche Diagnostic (Germany). TBS buffer was purchased from VWR (Sweden). The Tris-HCl pH7.4, NaCl, EDTA and Tween-20 stock solution were obtained from Karolinska Institutet substrate department (Sweden).

Western Blot

Following 24hpi and 48hpi infection with different doses of SARS-CoV-2, the cells were lysed in 2% SDS lysis buffer (50 mM Tris-Cl pH 7.4, 150 mM NaCl, 1 mM EDTA, 2% SDS, freshly supplemented with 1 mM DTT, 1x protease inhibitor cocktail and 1x phosphatase inhibitor cocktail) followed by boiling at 95°C for 10 min to inactivate the virus. The protein concentration was evaluated by DC Protein Assay from Bio-Rad (USA). Evaluation of protein expression was performed by running 20µg of total protein lysate on NuPage Bis Tris 4%-12% gels (Invitrogen, USA). Proteins were transferred using iBlot dry transfer system (Invitrogen, USA) and blocked for 1h using 5% milk or BSA in 0.1% TBS-t (0.1% Tween-20). Subsequent antibody incubations were performed at 4°C overnight or for 1h at room temperature followed by incubation for 1h at

room temperature with Dako polyclonal goat anti-rabbit or anti-mouse immunoglobulins/HRP (Agilent Technologies, USA). Membranes were washed using 0.1% TBS-T and proteins were detected using ECL or ECL Select (GE Healthcare, USA) on ChemiDoc XRS+ System (Bio-Rad Laboratories, USA). The Western blot analysis was performed by using antibodies targeting ACE2, TMPRSS2, β-Tubulin, RIG-I, MDA-5, TRIM25, ISG15, p-IRF3, p-STAT1 and GAPDH.

Scanning electron microscopy (SEM)

SEM for SARS-CoV-2 infected cells were performed as described previously (Szekely et al., 2020). Briefly, Cells grown on poly-L-lysin coated glass coverslips (Corning, United States) and 48h following infection the coverslips were fixed by immersion in 2.5% glutaraldehyde in 0.1M phosphate buffer, pH 7.4. The coverslips were rinsed with 0.1M phosphate buffer and Milli-Q® water prior to stepwise ethanol dehydration and critical-point-drying using carbon dioxide in an EM CPD 030 (Leica Microsystems, Germany). The coverslips were mounted on alumina specimen stubs using carbon adhesive tabs and sputter coated with a thin layer of platinum using a Q150T ES (Quorum Technologies, United Kingdom). SEM images were acquired using an Ultra 55 field emission scanning electron microscope (Carl Zeiss Microscopy GmbH, Germany) at 3kV and the SE2 detector.

Immunofluorescence and Confocal Microscopy

Calu-3 cells were grown on poly-L-lysin coated glass coverslips (Corning, United States). After 24h, 48h and 72h of incubation the cells were fixed in 4% paraformaldehyde (Merck, 100496). To stain endogenous β-actin and β-catenin, the cells were permeabilized with 0.05% Triton X-100 in PBS for 5 min at room temperature (RT), blocked with 3% BSA in PBST (0.05% Triton X-100 in PBS) for 30 mins, and labeled with rabbit anti-β-actin and mouse anti-β-catenin antibodies diluted in blocking buffer for 1h. Following primary antibody incubation, the coverslips were washed in PBS 3 times for 5 min each and then incubated with appropriate Alexa Fluor 488 and 568, conjugated secondary antibodies. The coverslips were mounted cell side down on object glasses with mounting medium containing DAPI to stain the nuclei. The samples were imaged using a confocal scanning laser microscope (Nikon ECLIPSE T*i*, Tokyo, Japan).

Quantitative proteomics analysis

Proteomics pipeline was performed similarly as we reported previously (Appelberg et al., 2020). Cell lines were divided in two different batches, batch 1: Calu-3 and Caco2 cells, and batch 2: 293FT and Huh7 cells. For each cell line in each batch, three biological replicates of mock-infected and SARS-CoV-2-infected cells (24h) were included. Briefly, proteins were extracted with SDS-based buffer, digestion was performed on S-Trap micro columns (Protifi, USA) and resulting peptides were labeled with isobaric TMTpro™ reagents. Labeled peptides were fractionated by high pH (HpH) reversed-phase chromatography and concatenated to a total of 12 fractions, of which each fraction was analyzed on an Ultimate 3000 UHPLC (ThermoFisher Scientific, USA) in a 120 min linear gradient. Data were acquired on a Orbitrap™ Q-Exactive HF-X™ mass spectrometer (ThermoFisher Scientific, USA) in data dependent acquisition (DDA) mode, isolating the top 20 most intense precursors at 120,000 mass resolution in the mass range of *m/z* 375 – 1400, and applying maximum injection time (IT) of 50 ms and dynamic exclusion of 30 s; precursor isolation width of 0.7 Th with high collision energy (HCD) of 34% at resolution of 45,000 and maximum IT of 86 ms in MS2 event.

Proteins were searched against both SwissProt human and SARS-CoV-2 (NCBI: txid2697049, downloaded on 2021-03-01) databases using the search engine Sequest HT in Proteome Discoverer v2.4 (ThermoFisher Scientific, USA) software environment allowing maximum two missed cleavages. Oxidation of methionine, deamidation of asparagine and glutamine, TMTpro modification of lysine and N-termini were set as variable modifications, while carbamidomethylation of cysteine was used as fixed modification. The false discovery rate (FDR) was set to 1%.

Data Availability

The raw mass spectrometric data was deposited to the ProteomeXchanger Consortium (http://proteomecentral.proteomexchange.org) via the PRIDE partner repository with the dataset identifier PXD023760. The unprocessed raw data files and processed data files are uploaded as excel files in Table S4 – Table S11. Additional Supplemental Items are available from Mendeley Data at. http://dx.doi.org/10.17632/tjr7cfhwm7.1.

Statistical analysis

Statistical analyses for proteomics and transcriptomics were performed in R package LIMMA. All other statistical calculations were performed in GraphPad Prism (Version 8.0.0) using unpaired t test. Significance values are indicated in the figures and figure legends. p*<0.05, **<0.01, ***<0.001 and ****<0.0001.

Bioinformatics analysis

Proteomics data of SARS-CoV-2 infected (moi 1) Huh7 (Appelberg et al., 2020) and Caco2 cells (Bojkova et al., 2020) were re-analyzed for this study. Differential abundance analysis was performed using R package LIMMA between mock and 48hpi for Huh7 and respectively mock and infected cells at 0, 6, 10, 24hpi. Pairwise comparisons were extracted and Benjamini-Hochberg (BH) adjustment was applied on p values. Genes with adjusted p values <0.05 were selected. Huh7, Caco2, Calu-3 and 293FT SARS-CoV-2 infected and mock infected cells were collected at 24h and TMT-labeled proteomics was performed. Proteomics raw data was first filtered for empty rows and quantile normalized with R package NormalizerDE. Histogram was used to display the distribution of data and assess that the distribution follows a normal law. Principal component analysis was performed using ggplot2 (supplemental table S12). The viral proteins were determined by matching the peptides to a database that was downloaded from NCBI on 1st March 2021, named as Severe acute respiratory syndrome coronavirus 2, identifier NCBI:txid2697049, consisting on 38 sequences. In order to measure the difference of abundance of viral proteins between cell lines we subtracted the value in uninfected cells (baseline) from the one in infected cells. Data was prior quantile normalized and followed normal distribution so we did not log2 transform the data. Viral protein abundances were retrieved and baseline subtraction (Infected-Mock) was performed for each time point and represented using barplots made with ggplot2. Differential abundance analysis was performed using LIMMA (Kammers et al., 2015). Three manually curated libraries based on interferon-regulated genes were created based on reactome terms "Antiviral mechanism by IFN−stimulated genes", "Interferon gamma signaling" and "Interferon alpha/beta signaling" (https://reactome.org/). Each library had respectively 89, 172 and 138 genes. The total number of interferon-regulated genes excluding overlap between libraries is 205 (supplemental table S13).

Protein profiles were represented as a heatmap using R ComplexHeatmap function. Venn diagrams were made using interactivenn (http://www.interactivenn.net/). Dotplot was made using ggplot2. Interferon-regulated significant proteins (LIMMA, FDR <0.05) were represented as a network with Cytoscape ver 3.6.1. For each node, fold changes were added to the network template file. Protein-protein interactions were retrieved from STRING Db (v5.0) (https://string-db.org/). Interactions were filtered on confidence score with minimum interaction of 0.700. Only interactions from databases and experiences were conserved.

R package ReactomePA was used for reactome pathway-based analysis. Pathways analysis results were represented using barplot enrichment maps available from the package. To compare biological pathways among several cell lines, R package clusterProfiler was used.

Supplemental references:

Appelberg, S., Gupta, S., Svensson Akusjarvi, S., Ambikan, A.T., Mikaeloff, F., Saccon, E., Vegvari, A., Benfeitas, R., Sperk, M., Stahlberg, M., *et al.* (2020). Dysregulation in Akt/mTOR/HIF-1 signaling identified by proteo-transcriptomics of SARS-CoV-2 infected cells. Emerg Microbes Infect *9*, 1748-1760.

Banerjee, A., Nasir, J.A., Budylowski, P., Yip, L., Aftanas, P., Christie, N., Ghalami, A., Baid, K., Raphenya, A.R., Hirota, J.A., *et al.* (2020). Isolation, Sequence, Infectivity, and Replication Kinetics of Severe Acute Respiratory Syndrome Coronavirus 2. Emerg Infect Dis *26*, 2054-2063.

Bojkova, D., Klann, K., Koch, B., Widera, M., Krause, D., Ciesek, S., Cinatl, J., and Munch, C. (2020). Proteomics of SARS-CoV-2-infected host cells reveals therapy targets. Nature *583*, 469-472.

Chu, H., Chan, J.F., Yuen, T.T., Shuai, H., Yuan, S., Wang, Y., Hu, B., Yip, C.C., Tsang, J.O., Huang, X., *et al.* (2020). Comparative tropism, replication kinetics, and cell damage profiling of SARS-CoV-2 and SARS-CoV with implications for clinical manifestations, transmissibility, and laboratory studies of COVID-19: an observational study. Lancet Microbe *1*, e14-e23.

Corman, V.M., Landt, O., Kaiser, M., Molenkamp, R., Meijer, A., Chu, D.K., Bleicker, T., Brunink, S., Schneider, J., Schmidt, M.L., *et al.* (2020). Detection of 2019 novel coronavirus (2019-nCoV) by real-time RT-PCR. Euro Surveill *25*.

Hsin, F., Chao, T.-L., Chan, Y.-R., Kao, H.-C., Liu, W.-D., Wang, J.-T., Pang, Y.-H., Lin, C.-H., Tsai, Y.-M., Lin, J.-Y., *et al.* (2020). Distinct Inductions of and Responses to Type I and Type III Interferons Promote Infections in Two SARS-CoV-2 Isolates. bioRxiv.

Jureka, A.S., Silvas, J.A., and Basler, C.F. (2020). Propagation, Inactivation, and Safety Testing of SARS-CoV-2. Viruses *12*.

Kammers, K., Cole, R.N., Tiengwe, C., and Ruczinski, I. (2015). Detecting Significant Changes in Protein Abundance. EuPA open proteomics *7*, 11-19.

Liao, Y., Li, X., Mou, T., Zhou, X., Li, D., Wang, L., Zhang, Y., Dong, X., Zheng, H., Guo, L., *et al.* (2020). Distinct infection process of SARS-CoV-2 in human bronchial epithelial cells line. J Med Virol.

Matsuyama, S., Nao, N., Shirato, K., Kawase, M., Saito, S., Takayama, I., Nagata, N., Sekizuka, T., Katoh, H., Kato, F., *et al.* (2020). Enhanced isolation of SARS-CoV-2 by TMPRSS2-expressing cells. Proc Natl Acad Sci U S A *117*, 7001-7003.

Rohde, C., Becker, S., and Krahling, V. (2019). Marburg virus regulates the IRE1/XBP1-dependent unfolded protein response to ensure efficient viral replication. Emerg Microbes Infect *8*, 1300-1313.

Szekely, L., Bozoky, B., Bendek, M., Ostad, M., Lavignasse, P., Haag, L., Wu, J., Jing, X., Gupta, S., Saccon, E., *et al.* (2020). Pulmonary stromal expansion and intra-alveolar coagulation are primary causes of Covid-19 death. bioRxiv, 2020.2012.2023.424172.

Wurtz, N., Penant, G., Jardot, P., Duclos, N., and La Scola, B. (2020). Culture of SARS-CoV-2 in a panel of laboratory cell lines. IHU.

Zecha, J., Lee, C.Y., Bayer, F.P., Meng, C., Grass, V., Zerweck, J., Schnatbaum, K., Michler, T., Pichlmair, A., Ludwig, C., *et al.* (2020). Data, Reagents, Assays and Merits of Proteomics for SARS-CoV-2 Research and Testing. Mol Cell Proteomics *19*, 1503-1522.
